# Effectiveness of Single-Tablet Combination Therapy in Improving Adherence and Persistence and the Relation to Clinical and Economic Outcomes

**DOI:** 10.36469/001c.91396

**Published:** 2024-01-23

**Authors:** Carly J. Paoli, Jörg Linder, Khushboo Gurjar, Deepika Thakur, Julie Wyckmans, Stacy Grieve

**Affiliations:** 1 Janssen Pharmaceutical Companies of Johnson & Johnson, Titusville, New Jersey, USA; 2 Janssen-Cliag of Johnson & Johnson, Neuss, Germany; 3 Cytel Inc., Waltham, Massachusetts, USA; 4 Janssen Pharmaceutical Companies of Johnson & Johnson, Basel, Switzerland

**Keywords:** single-tablet combination therapy, fixed-dose combination, loose-dose combination, cost-effectiveness, real-world outcomes

## Abstract

**Background:** Single-tablet combination therapies (STCTs) combine multiple drugs into one formulation, making drug administration more convenient for patients. STCTs were developed to address concerns with treatment adherence and persistence, but the impact of STCT use is not fully understood across indications.

**Objectives:** We conducted a systematic literature review (SLR) to examine STCT-associated outcomes across 4 evidence domains: clinical trials, real-world evidence (RWE), health-related quality of life (HRQoL) studies, and economic evaluations.

**Methods:** Four SLRs were conducted across the aforementioned domains. Included studies compared STCTs as well as fixed-dose combinations ([FDCs] of non-tablet formulations) with the equivalent active compounds and doses in loose-dose combinations (LDCs). Original research articles were included; case reports, case series, and non-English-language sources were excluded. Databases searched included EconLit, Embase, and Ovid MEDLINE® ALL. Two independent reviewers assessed relevant studies and extracted data. Conflicts were resolved with a third reviewer or consensus-based discussion.

**Results:** In all, 109 studies were identified; 27 studies were identified in more than one SLR. Treatment adherence was significantly higher in patients receiving FDCs vs LDCs in 12 of 13 RWE studies and 3 of 13 clinical trials. All 18 RWE studies reported higher persistence with FDCs. In RWE studies examining clinical outcomes (n = 17), 14 reported positive findings with FDCs, including a reduced need for add-on medication, blood pressure control, and improved hemoglobin A1C. HRQoL studies generally reported numerical improvements with STCTs or similarities between STCTs and LDCs. Economic outcomes favored STCT use. All 6 cost-effectiveness or cost-utility analyses found FDCs were less expensive and more efficacious than LDCs. Four budget impact models found that STCTs were associated with cost savings. Medical costs and healthcare resource use were generally lower with FDCs than with LDCs.

**Discussion:** Evidence from RWE and economic studies strongly favored STCT use, while clinical trials and HRQoL studies primarily reported similarity between STCTs and LDCs. This may be due to clinical trial procedures aimed at maximizing adherence and HRQoL measures that are not designed to evaluate drug administration.

**Conclusions:** Our findings highlight the value of STCTs for improving patient adherence, persistence, and clinical outcomes while also offering economic advantages.

## INTRODUCTION

Chronic diseases result in high healthcare resource utilization (HCRU) and substantial costs for healthcare systems. Patients with chronic diseases frequently have low treatment adherence and persistence rates, which can result in poor clinical outcomes and contribute to higher HCRU and costs.^1–6^ Poor adherence can be driven by various factors, including a lack of health literacy, comorbidities and polypharmacy, inadequate access to medical care, and the high cost of treatments.^7^ Mechanisms to improve patient adherence and persistence are increasingly sought after to advance the efficiency of healthcare systems and produce better health outcomes for patients,^7–9^ particularly patients with chronic diseases where combination therapy is recommended by clinical guidelines.^10–12^ Single-tablet combination therapies (STCT) that combine multiple drugs into one formulation may address some of the reasons for poor adherence by reducing the pill burden for patients with chronic diseases. Previous work has shown that across multiple indications, STCTs can encourage treatment adherence^13^ and persistence,^14,15^ and provide economic evidence supporting the use of STCTs for reducing costs and HCRU.^16,17^

The number of STCTs that have received regulatory approval in the United States (US) and Europe has increased in recent years. In the US, STCT approvals rose from 12 approvals in the 1980s to 59 approvals in the 2000s,^18^ while in Europe, 7 STCTs were approved in 2016, compared with just 1 in 2010.^19^ STCTs are now available for hypertension, HIV, asthma, diabetes, and other chronic diseases. The evaluation of STCT use is relevant to multiple stakeholders, including patients, clinicians, caregivers, and payers. To accurately assess the value provided by STCTs, a comprehensive picture of their impact is needed across indications, countries, and types of evidence. The goal of this systematic literature review (SLR) was to characterize the effects of STCTs and loose-dose combination products (LDC) on treatment adherence, compliance, persistence, clinical outcomes, economic outcomes, and health-related quality of life (HRQoL) across 4 evidence domains: clinical trials, real-world evidence (RWE), HRQoL studies, and economic evaluations.

## METHODS

Four SLRs were conducted with unique search strategies to identify the most relevant records under the aforementioned research domains (**Supplemental Table 1**). Database and registry records published between January 2001 and December 2021 that compared fixed-dose combinations and LDCs were searched according to Preferred Reporting Items for Systematic Reviews and Meta-Analyses (PRISMA) guidelines.^20^ Only comparisons of fixed-dose combinations and LDCs with the same active compounds and doses were considered. The databases searched included Cochrane, American College of Physicians Journal Club, National Health Service Economic Evaluation Database, EconLit, Embase, and Ovid MEDLINE® ALL. Case reports, case series, and non-English language records were excluded according to the population, intervention, comparator, outcomes and study design criteria (**Supplemental Table 2**). Two independent reviewers identified relevant studies at the title and abstract level, assessed the full text of included studies, and extracted relevant study data. A third reviewer or consensus-based discussion was used to resolve any conflicts that arose during the screening and data extraction process. Data were extracted to Microsoft Excel® [v.2301]. Figures and tables were created using Microsoft Excel® [v.2301] and PowerPoint^®^ [v.2301].

Real-world and clinical trial evidence are associated with different limitations and advantages for assessing treatment adherence and its effect on clinical outcomes. Clinical trials occur in a controlled setting with protocols to ensure that a direct comparison can be made between study arms. Patient behavior is closely monitored to ensure that patients take medication as prescribed, resulting in higher adherence than would be observed outside of the clinical trial environment. Additionally, patient populations in clinical trials are often more homogenous than in real-world settings.^21^ In contrast, RWE is based on real-world data, often collected as part of routine healthcare administration and billing. Patients are not randomized and may be assigned to treatments based on physician bias, patient request, or insurance coverage.^22^ Patient behaviors are typically captured more accurately by RWE. We chose to seek out both clinical trial data and RWE, since information from these sources is often complementary to one another.

The SLRs of RWE and clinical trial data identified treatment adherence, compliance, and persistence outcomes. The terms, while similar, denote different aspects of patient behavior as it relates to clinical recommendations and prescriptions. Adherence is defined as the proportion of prescribed pills taken over a specific interval of time.^23^ While the adherence threshold can vary across medications, a patient is generally considered adherent if they align with their prescribed dosing schedule 80% of the time.^24^ Commonly used measures of adherence report the proportion of prescribed medication that a patient acquired at the pharmacy; these include the medication possession ratio (MPR) and the proportion of days covered.^25^ In contrast to adherence, compliance encompasses a broader definition, referring to the extent to which patients align their medication usage with the recommended dosage, timing, and frequency provided by healthcare professionals in their day-to-day clinical management.^26^ Treatment persistence refers to the act of continuing to take clinically recommended medication.^26^ In the literature, some research studies use the terms *treatment adherence* and *treatment compliance* interchangeably. No subjective decisions were made to recategorize the data in identified studies; instead, the measures were reported as they were originally described. The number of studies as reported in the results are not mutually exclusive as some studies report multiple outcomes.

When possible, comparable studies that reported the same outcome measures were placed in context with one another for data visualization and reporting, but no data transformations or meta-analyses were conducted. Outcome measures were included when the same metrics were reported across fixed-dose combination and LDC versions of equivalent formulations and doses, either from clinical trials, cohort studies, retrospective database studies, or switching studies, in which patients on LDCs with baseline measurements were switched to treatment with equivalent fixed-dose combinations. Statistically significant and numerical findings were reported as described in each study; no post-hoc statistical tests were performed.

## RESULTS

**Figure 1.**
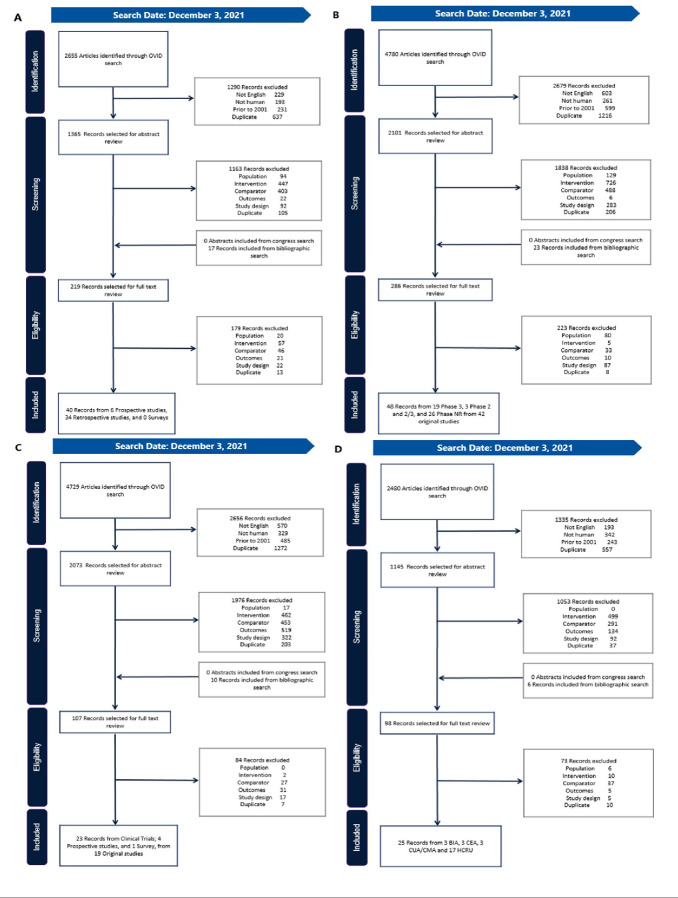
Preferred Reporting Items for Systematic Reviews and Meta-Analyses Diagrams Our findings included evidence relating to treatment adherence, treatment persistence, and clinical outcomes in RWE (**A**, n = 40) and clinical trials (**B**, n =48), along with HRQoL (**C**, n = 23) and economic outcomes (**D**, n = 25). Abbreviations: BIA, budget impact analysis; CEA, cost-effectiveness analysis; CMA, cost-minimization analysis; CUA, cost-utility analysis; HRQoL, health-related quality of life; HCRU, healthcare resource utilization.

After accounting for duplicate records, 109 original studies were included in our findings (**Figure 1**).^27–134^ Twenty-seven of these studies were identified in more than 1 SLR.^33,34,37,40,42,44,47,48,51–53,55,65–68,75,87,98,100,106–108,116,119,128,133^ A list of the identified studies by SLR, along with study type and the indications included, is presented in **Supplemental Table 3**. North American, European, and Asian countries were well represented in studies relating to clinical evidence, HRQoL, and economic outcomes, while RWE was reported only from some North American and European countries.

STCTs were the most common fixed-dose formulation (n = 72), followed by inhaled suspensions (n = 18), eye-drop solutions (n = 16), and injected solution and nasal spray (n = 1 each).

### Treatment Adherence, Compliance, Persistence, and Clinical Outcomes in RWE

Forty RWE studies (n = 34 retrospective observational^31,33,36,38,40,42,47,54–56,58,59,74,76,85–88,94,96,97,99,100,110–115,121,122,126,127,129^ and n = 6 prospective observational)^49,63,65,71,103,104^ reported information on adherence rates, persistence rates, compliance, or clinical outcomes. The included clinical indications were frequently those treated with multiple drugs, and/ or with common comorbidities that added to the pill burden, such as cardiovascular disease (n = 23, including 19 hypertension),^40,42,49,54,59,63,71,74,76,86–88,94,96,97,99,100,110–115^ type 2 diabetes mellitus (n = 10),^31,36,38,47,55,58,103,104,126,127^ HIV (n = 3),^33,85,129^ asthma (n = 2),^121,122^ and 1 study each for chronic obstructive pulmonary disease (COPD)^65^ and Parkinson’s disease.^56^

Thirteen studies reported treatment adherence in patients taking STCTs vs LDCs, using MPR (n = 11), proportion of days covered (n = 7), or mean adherence (n = 3). In 12 of those studies, treatment adherence was significantly higher in patients who received fixed-dose combinations compared with LDCs^36,40,42,55,56,76,85–87,96,97,122^; in the remaining study, patients taking STCTs had a numerically higher adherence.^49^ An additional 8 studies described treatment adherence in patients who switched from LDCs or monotherapy to fixed-dose combinations,^31,38,47,54,58,71,126,127^ with reported follow-up times ranging from 3 months^63^ to 36 months.^38^ Patients who switched to STCTs had higher treatment adherence in all studies except for one, in which patients switched from sulfonylureas (including glyburide) plus metformin to glyburide/ metformin for type 2 diabetes.^58^ Even in this study, the STCT had better clinical outcomes (improvement of glycemic control, A1C reduction of 0.6% (*p* = .002), and the difference in adherence was not statistically significant (92.4% vs 90.9% after the switch to STCT). Of the studies that reported improved adherence with STCTs, 8 were of patients with hypertension^40,42,49,54,86,87,96,97^ and 6 were of patients with type 2 diabetes mellitus.^31,36,38,47,55,126^

Four studies reported treatment compliance with STCTs or fixed-dose inhalers compared with LDCs.^65,103,104,110^ Treatment compliance was significantly lower in patients taking LDCs in 2 of the 4 studies.^103,104^ In the remaining 2 studies, compliance was numerically high with both fixed-dose combinations and LDCs (70.0%-80.0%),^110^ and similar in 1 study using a fixed-dose inhaler (compliance defined as residual doses in the inhalation device returned by the patient 97.1% in the fixed dose inhaler group vs 98.4% in the 2-inhaler group).^65^

Of the 18 studies that examined treatment persistence, all studies reported higher persistence in patients taking STCTs or fixed-dose inhalers compared with LDCs. Among these 18 studies, 16 reported significantly higher persistence in patients taking STCTs or fixed-dose inhalers compared with LDCs in 1 or more treatment arms^40,42,56,59,74,87,96,97,100,110,112–115,121,122^; while the remaining 2 studies did not report statistical significance, only a numerically higher treatment persistence with STCTs.^88,111^ Thirteen studies reported mean 1-year persistence in STCTs vs LDCs (**Table 1**)^40,42,59,74,87,88,96,110–115^; of these, 9 studies reported ≥20% higher persistence in patients taking STCT.^42,59,74,87,96,111–113,115^ When summarized across studies, mean 1-year persistence was 51.9% for STCTs and 31.5% for LDCs. In a large retrospective study that included 81 958 patients with hypertension, treatment persistence ranged from 16% to 42% greater in patients on STCTs compared with LDCs.^40^

**Table 1.** Summary of RWE Findings for Persistence, Compliance, and Adherence, Where Comparable

**Source**	**Indication**	**Population**	**Intervention**	**STCT**	**LDC**	**ΔSTCT–⁠LDC**
**Mean 1-year persistence (%)**						
Bramlage et al, 2018^40^	HTN	Patients with HTN	Ramipril/amlodipine	65.7	48.6	17.10
			Candesartan/amlodipine	55.5	43.1	12.40
Brixner et al, 2008^42^	HTN	Adults with HTN	Valsartan/ hydrochlorothiazide	44.0	16.0	28.00
Ehlken et al, 2011^59^	HTN	Patients with HTN	Olmesartan/ hydrochlorothiazide	44.6	25.0	19.60
			Olmesartan/amlodipine	47.3	27.4	19.90
			Valsartan/ hydrochlorothiazide	39.6	13.7	25.90
			Valsartan/amlodipine	44.6	25.2	19.40
Jackson et al, 2006^74^	HTN	Adults with HTN	Valsartan/ hydrochlorothiazide	44.0	16.0	28.00
Machnicki et al, 2015^87^	HTN	Adults with HTN	Amlodipine/valsartan/ hydrochlorothiazide	46.8	23.6	23.20
Maggioni et al, 2019^88^	ACS	Patients with ACS	Aspirin/clopidogrel	81.5	72.9	8.60
Ong et al, 2014^96^	HTN	Patients with HTN	Amlodipine/valsartan/ hydrochlorothiazide (Exforge) HCT)	47.2	23.6	23.60
Sandberg et al, 2011^110^	HTN	Patients with HTN	Olmesartan/ hydrochlorothiazide	44.6	25.0	-19.6
			Olmesartan/amlodipine	47.3	27.4	19.9
Simons et al, 2011^112^	HTN	Patients with HTN	Amlodipine/atorvastatin	67.0	41.0	26.00
Simons et al, 2017^111^	HTN	Patients with HTN	Amlodipine/atorvastatin	66.0	43.0	23.00
Simonyi et al, 2015^113^	HTN	Patients with HTN	Ramipril/amlodipine	54.0	34.0	20.00
Simonyi et al, 2016^114^	HTN	Patients with HTN	Perindopril/amlodipine	40.0	27.0	13.00
Simonyi et al, 2017^115^	HTN	Patients with HTN	Ramipril/amlodipine	54.0	34.0	20.00
**Mean compliance (%)**						
Hagedorn et al, 2013^65^	COPD	Adults with stage 3 or 4 COPD	Fluticasone/salmeterol	97.1	98.4	-1.30^a^
Rombopoulos et al, 2012^104^	T2DM	Adults with T2DM	Vildagliptin/metformin	68.0	56.0	12.00
Rombopoulos et al, 2015^103^	T2DM	Adults with T2DM	Vildagliptin/metformin	98.9	84.6	14.30
**Mean adherence (%)**						
Degli et al, 2018^54^	HTN	Adult with HTN	Perindopril + amlodipine to perindopril/amlodipine	79.8	70.9	8.90
Duckworth, 2003^58^	T2DM	Adults with T2DM who had been treated with glipizide or glyburide plus metformin 6 mo prior to switching to glyburide/ metformin	Glyburide + metformin or glipizide + metformin to glyburide/metformin	90.9	92.4	-1.50^b^
Legorreta et al, 2005^85^	HIV	Adults with HIV who initiated therapy on or after September 1997	Lamivudine/zidovudine	85.0	75.0	10.00

^a^Statistical significance not measured.

^b^No statistically significant difference.

Abbreviations: ACS, acute coronary syndrome; COPD, chronic obstructive pulmonary disease; HTN, hypertension; LDC, loose-dose combination; STCT, single-tablet combination therapy; T2DM, type 2 diabetes mellitus.

Clinical outcomes were reported by 17 studies, across indications of asthma, COPD, hypertension, and type 2 diabetes mellitus (**Table 2**).^36,40,42,49,58,63,65,71,94,99,100,103,104,111,121,122,126^ Positive findings with STCTs or fixed-dose inhalers were reported in all but 3 studies that described similar outcomes with STCTs/fixed-dose inhalers and LDCs.^65,104,121^ Of the studies that reported improved clinical outcomes with STCTs, 3 contained caveats that may weaken the finding, such as the likelihood of residual confounders,^36,94,111^ and 2 reported improvements in subgroups of adherent or persistent patients only.^42,94^ Significantly improved clinical outcomes included hemoglobin A1C (n = 3),^36,58,126^ the need for add-on medication (n = 3),^40,42,122^ all-cause hospitalization (n = 2),^99,100^ blood pressure control (n = 1),^63^ and all-cause mortality (n = 1).^99^ Six studies reported statistical significance in both clinical outcome improvement and adherence, compliance, or persistence improvement.^40,42,100,103,122,126^

**Table 2.** Reported Clinical Outcomes With STCTs

**Source**	**Intervention**	**Clinical Outcome Compared With LDC**	**Adherence, Compliance, and/or Persistence Compared With LDC**
**Asthma studies**			
Stempel et al, 2005^121^	Ramipril/amlodipine	No difference in the need for add-on SABA medication	Persistence improvement^a^
Stoloff et al, 2004^122^	Fluticasone/salmeterol	Reduced need for add-on SABA medication^a^	Adherence improvement^a^ Persistence improvement^a^
**Cardiovascular disease studies**			
Ofili et al, 2017^94^	Isosorbide dinitrate/ hydralazine hydrochloride^c^	Among adherent Black American patients, improved 1-year overall survival^a^	Worsened adherence
Predel et al, 2020^100^	Aspirin/ramipril/ atorvastatin	Reduced incidence rate ratios for: All-cause hospitalization^a^ All-cause mortality Coronary artery disease Myocardial infarction Stroke Transitory ischemic attack	Persistence improvement^a^
Predel et al, 2021^99^	Ramipril/amlodipine	Reduced incidence rate ratios for: All cause hospitalization^a^ Cardiovascular hospitalization^a^ All-cause mortality^a^ Coronary artery disease^a^ Heart failure^a^ Myocardial infarction^a^ Stroke^a^ Transitory ischemic attack^a^	Not measured
**COPD**			
Hagerdorn et al, 2013^65^	Fluticasone/salmeterol	No difference in: FEV_1_ IVC Tiffeneau index Number of exacerbations Use of rescue medication	No difference in compliance
**Hypertension studies**			
Bramlage et al, 2018^40^	Ramipril/amlodipine	Smaller improvement in SBP compared with LDC No difference in DBP Reduced need for add-on medication^a^	Adherence improvement^a^ Persistence improvement^a^
	Candesartan/ amlodipine	SBP improvement DBP improvement Reduced need for add-on medication^a^	Adherence improvement^a^ Persistence improvement^a^
Brixner et al, 2008^42^	Valsartan/ hydrochlorothiazide	Among persistent patients, those receiving an STCT had a reduced need for add-on medication^a^	Adherence improvement^a^ Persistence improvement^a^
Czarnecka et al, 2015^49^	Bisoprolol/amlodipine	SBP improvement DBP improvement	Adherence improvement
Gaciong et al, 2017^63^	Bisoprolol/ acetylsalicylic acid	SBP improvement^a^ DBP improvement^a^ Heart rate improvement^a^	Not measured
Hostalek et al, 2015^71^	Bisoprolol/amlodipine	SBP improvement DBP improvement Heart rate improvement	Not measured
Simons 2017^111^	Amlodipine/ perindopril	Reduced risk of mortality^a,d^	Persistence improvement
**T2DM studies**			
Blonde et al, 2003^36^	Glyburide/metformin	A1C improvement^a^	Adherence improvement^b^
Duckworth 2003^58^	Glyburide/metformin	A1C improvement^a^	Worsened persistence
Rombopoulos et al, 2012^104^	Vildagliptin/ metformin	No difference in A1C	Compliance improvement^a^
Rombopoulos et al, 2015^103^	Vildagliptin/ metformin	A1C improvement	Compliance improvement^a^
Thayer et al, 2010^126^	Rosiglitazone/ sulfonylurea	A1C improvement^a^	Adherence improvement^a^

^a^Statistically significant result.

^b^Linear regression did not find a relationship between adherence and improved A1C.

^c^STCT may not be bioequivalent to its LDC comparator.^14^

^d^Study authors attributed result to residual confounders.

Abbreviations: A1C, hemoglobin A1C; COPD, chronic obstructive pulmonary disease; DBP, diastolic blood pressure; FEV_1_, forced expiratory volume in 1 second; IVC, inspiratory vital capacity; LDC, loose-dose combination therapy; SABA, short- acting β-agonist; SBP, systolic blood pressure; STCT, single-tablet combination therapy; T2DM, type 2 diabetes mellitus.

### Treatment Adherence, Compliance, Persistence, and Clinical Outcomes in Clinical Trials

The SLR for clinical trial evidence identified 48 records from 42 studies across the indications of glaucoma/ocular hypertension (n = 10),^30,35,57,61,66–69,81,134^ hypertension (n = 6),^41,60,83,92,124,131^ asthma (n = 7),^37,72,75,98,106,107,133^ tuberculosis (n = 6),^28,32,79,91,123,125,132^ HIV (n = 3),^48,108,128^ and others. Twenty-six studies did not identify the study phase,^28,29,32,34,44,50,57,60,61,75,80,81,83,92,93,98,106–108,123–125,128,131–133^ 14 were Phase 3 studies,^27,30,35,37,39,41,48,51–53,66–69,72,82,89,116,134^ 1 was Phase 2,^45^ and 1 was Phase 2/3.^79,91^ Clinical trials were geographically diverse, with more studies including participants from Africa and Asia compared with the other study types. Thirteen studies reported adherence outcomes with fixed-dose combinations and LDCs: 9 reported similar outcomes with fixed-dose combinations and LDCs,^28,48,75,80,89,92,98,108,133^ 3 reported a statistically significant improvement in adherence with STCTs,^45,83,124^ and 1 reported a numerical improvement with fixed-dose eye drops.^30^ Among the studies that reported a significant improvement in adherence with STCTs, 2 also reported improvements in blood pressure control.^83,124^ All 4 studies that described compliance outcomes reported similar findings with STCTs/fixed-dose inhalers and LDCs.^27,28,37,123^

### Health-Related Quality of Life Findings

HRQoL findings were reported in 23 records of 19 studies,^34,37,44,48,51–53,64–68,73,75,95,98,106–108,116,119,128,133^ which included 14 clinical trials^34,37,44,48,51–53,66–68,75,98,106–108,116,128,133^ and 5 observational studies^64,65,73,95,119^ primarily from North American and European countries. The studies included indications consisting of asthma (n = 7),^37,75,98,106,107,119,133^ HIV (n = 3),^48,108,128^ COPD (n = 2),^51–53,65^ and glaucoma (n = 2),^66–68,73^ among others. The study findings were largely noncomparable due to the heterogeneous methods used to assess HRQoL, which included generic and disease-specific scales, patient-reported outcome measures, and symptom reporting. Findings from the studies were mixed, with 5 of 8 treatment arms in the observational studies reporting a numerical (n = 4)^64,65,73,95^ or significant (n = 1)^64^ improvement in HRQoL with STCTs, and the remaining 3 treatment arms describing similar outcomes with fixed-dose combinations and LDCs.^65,73,119^ Results were similar between fixed-dose combinations and LDCs in the majority (n = 9)^34,44,48,53,68,98,107,128,133^ of the interventional HRQoL studies, but numerical improvements with fixed-dose combinations (n = 3)^37,108,116^ and LDCs (n = 1)^75^ were also reported.

### Economic Outcomes

Economic outcomes were evaluated in 25 studies from 11 countries in North and South America, Europe, and Asia, and reported on indications of hypertension (n = 10),^40,42,46,62,70,77,78,87,102,120^ asthma (n = 4),^43,90,105,119^ diabetes (n = 3),^47,55,109^ cardiovascular disease (n = 3),^100,117,118^ COPD (n = 2),^65,101^ glaucoma/ocular hypertension (n = 2),^84,130^ and HIV (n = 1).^33^ Six cost-effectiveness or cost-utility analyses compared STCT or fixed-dose inhalers with LDCs (n = 6, **Table 3**),^43,62,77,101,102,120^ including 3 branded STCTs^62,77,101^ and 1 branded fixed-dose inhaler.^43^ All 6 analyses found that fixed-dose combinations were both less expensive and more efficacious than LDCs (in economic terminology, fixed-dose products dominated LDCs). Several budget impact analyses evaluated the use of STCTs and LDCs for patients with cardiovascular diseases, including hypertension and hyperlipidemia (n = 4, **Table 4**).^62,78,117,118^ The scope of the analyses varied, with some studies estimating the cost savings from public payer^62,78^ and patient perspectives^78^ and others calculating the costs of medication^117^ or treatment.^118^ In all 4 studies, STCTs were associated with savings compared with LDCs.

**Table 3.** Findings from Cost-Effectiveness Models and Cost-Utility Analyses

**Reference**	**Study Design**	**Country**	**Population**	**Intervention (STCT Brand)**	**Main Message**
Kawalec et al, 2015^77^	CEA + CUA	Poland	Patients with arterial hypertension	Indapamide + amlodipine (Tertens-AM)	STCT was less expensive than treatment with LDCs
			Subgroup: STCT patients with higher adherence		STCT dominates LDCs, in both NHF and patient’s perspective
Ren et al, 2020^102^	CEA + CUA	China	Hypertensive adults	Olmesartan + amlodipine (Sevikar)	STCT dominates LDCs
Fujii et al, 2015^62^	CEA, BIA	Brazil	Systemic arterial hypertension	Bisoprolol + amlodipine (Concor AM)	STCT dominates LDCs
Stawowczyk et al, 2014^120^	CUA	Poland	Hypertensive patients	Indapamide + amlodipine (Natrixam)	**Public payer perspective:** STCT dominates LDCs **Patient’s perspective:** Net monetary benefit was higher with STCT
Price et al, 2014^101^	CMA	Sweden	Moderate-to-severe COPD patients	Indacaterol + glycopyrronium (Utibron)	STCT dominates LDCs in all horizons, and in PSA iterations
Brüggenjürgen et al, 2010^43^	CMA	Germany	Asthma	Beclomethasone dipropionate + formoterol fumarate (CHF 1535)	STCT dominates LDCs

Note: The result of the CEA from Fujii et al (2015) is shown here; findings from the BIA from Fujii et al (2015) is reported in **Table 4**.

Abbreviations: BIA, budget impact analysis; CEA, cost-effectiveness analysis; CMA, cost-minimization analysis; COPD, chronic obstructive pulmonary disease; CUA, cost-utility analysis; LDC, loose-dose combination; NHF, National Health Fund; PSA, probabilistic sensitivity analysis; STCT, single-tablet combination therapy.

**Table 4.** Findings from Budget Impact Analyses

**Short Reference**	**Country; Currency**	**Time Horizon**	**Population**	**Intervention (STCT** **Brand)**	**Savings**	**Budget Impact**	**Main Message**
Fujii et al, 2015^62^	Brazil; Brazilian real (BRL)	10 y	Systemic arterial hypertension	Bisoprolol + amlodipine (Concor AM)	NR	-300 321 412	There were financial resource savings with Concor AM, in 2014- 2025
Kawalec et al, 2014^78^	Poland; Polish zloty (PLN)	3 y	Hypertensive patients	Indapamide + amlodipine (Natrixam)	Cost savings at year 3: **NHF** **perspective:** -725 965 **Patient perspective:** -8 328 480	NR	Treatment with indapamide/amlodipine STCTs vs LDCs generates significant savings both from the public payer and patient perspectives
Stafylas et al, 2018^117^	Greece; euros (€)	5 y	Essential hypertension and/or stable coronary artery disease in association with primary hypercholesterolemia or mixed hyperlipidemia	Atorvastatin + perindopril + amlodipine	Year 5: -1 903 639	Cumulative over 5 y: -6 107 965	Introduction of STCTs at a price lower than that of the LDCs of the same agents will reduce medication costs
Stafylas et al, 2019^118^	Greece; euros (€)	5 y	Hyperlipidemia patients	Rosuvastatin + ezetimibe	Year 5: -395 447	Cumulative over 5 y: -2 351 952	The introduction of STCTs of ezetimibe/ rosuvastatin is expected to reduce treatment costs

Note: The result of the BIA from Fujii et al (2015) is shown here; findings from the CEA from Fujii et al (2015) is reported in **Table 3**.

Abbreviations: BIA, budget impact analysis; CEA, cost-effectiveness analysis; COPD, chronic obstructive pulmonary disease; CUA, cost-utility analysis; LDC, loose-dose combination; NHF, National Health Fund; NR, not reported; PSA, probabilistic sensitivity analysis; STCT, single-tablet combination therapy

**Figure 2.**
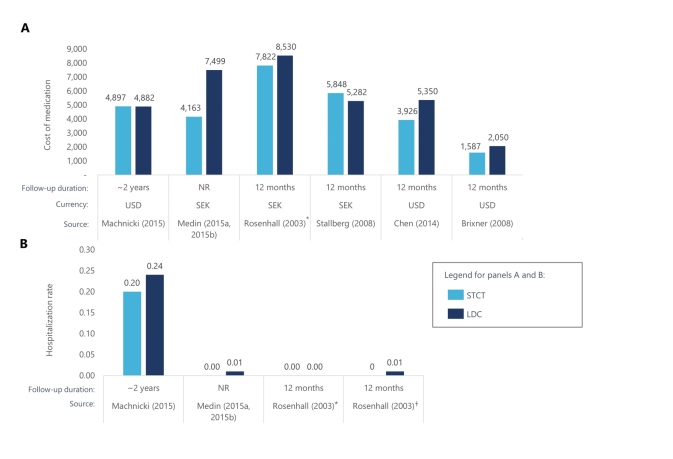
Medication Costs for STCTs and LDCs Several of the identified economic studies reported the cost of medication over time for STCTs vs LDCs. Of these, 3 studies also reported hospitalization rates for STCTs vs LDCs. *Patients in this comparison received budesonide + formoterol in a single inhaler or 2 inhalers †Patients in this comparison received budesonide + formoterol + terbutaline PRN in 2 or 3 inhalers Abbreviations: LDC, loose-dose combination product; NR, not reported; SEK, Swedish krona; STCT, single-tablet combination therapy; USD, US dollar. Sources: References 42, 46, 87, 90, 105, 119.

Among the 16 studies that examined medical costs and HCRU, findings were largely favorable toward fixed-dose combinations.^33,40,42,46,47,55,65,70,84,87,90,100,105,109,119,130^ Medication costs over time were generally lower for fixed-dose combinations compared with LDCs (n = 6,^42,46,87,90,105,119^ **Figure 2A**). Several studies reported lower overall medical costs with STCTs,^42,46,87^ including a retrospective cohort study that included 8711 US patients with hypertension.^42^ Patients received valsartan plus hydrochlorothiazide as an STCT or LDC and HCRU costs were compared after 12 months.^42^ The average annual medical costs for patients receiving an STCT were $474 lower than for patients who received an LDC during the study period of 1996 to 2004. When patients were both persistent and adherent to medication, medical costs were $608 lower. There was also evidence of STCTs and fixed-dose inhalers reducing HCRU, with 3 studies reporting lower hospitalization rates over time (n = 3).^87,90,105^ STCTs were also associated with reductions in emergency department costs^87^ and utilization rates.^46,87,100^

## DISCUSSION

The results of this study reflect the growing body of evidence that link STCTs to increased treatment adherence and persistence, along with positive clinical and economic outcomes. The strongest evidence supporting STCT use came from the RWE and economic studies. Improved adherence and persistence with STCTs were seen consistently across RWE studies, including studies where patients switched from an LDC to an STCT. The majority of the studies that reported clinical outcomes with STCTs described improvements, particularly in glycemic control and the reduced need for add-on medication. Several studies linked improved adherence or persistence outcomes with improved clinical efficacy. Studies that did not find a link between adherence/persistence and clinical outcomes included patients with complex, chronic diseases, such as asthma, COPD, and cardiovascular disease. It is possible that unmeasured confounders, such as disease severity, time since diagnosis, or symptom presentation affected the measurement of clinical outcomes, and more work is needed to better describe the impact of STCTs for patients with these indications. Only 4 studies were identified that described compliance outcomes, perhaps due to evolving terminology that increasingly favors “adherence” over “compliance,” and retrospective studies that often include adherence and persistence measures based on patient records. The economic findings in favor of STCTs were robust and suggested that the improved clinical outcomes associated with STCTs can lead to reduced medication costs, hospitalizations, and overall medical costs over time. All the identified cost-effectiveness, cost-utility, and budget impact analyses demonstrated savings with STCTs compared with LDCs.

While some clinical and HRQoL studies reported improvement in adherence and clinical outcomes with STCTs, the results were more heterogeneous. This may be a result of data collection methodology that is not optimized to compare outcomes with STCTs and LDCs. The controlled environment of clinical trials is designed to facilitate high treatment adherence, which may increase the adherence and persistence outcomes for LDCs and affect LDC-STCT comparisons in ways unrelated to pill burden. In studies that reported HRQoL outcomes, there was some evidence of improved outcomes among patients who received STCTs compared with those who received LDCs. However, most studies focused primarily on patient symptoms and general measures of HRQoL, rather than pill burden or drug administration processes. There is a need for future studies to compare STCTs and LDCs with HRQoL measures that more closely reflect the aspects of drug administration. Since there is evidence linking poor treatment satisfaction to poor adherence in patients with chronic diseases,^135,136^ validated tools to measure treatment satisfaction, such as the Treatment Satisfaction Questionnaire for Medication^137^ and the Diabetes Medication System Rating Questionnaire,^138^ may be useful in understanding how STCTs can affect HRQoL.

Our results, which included studies from countries on 6 continents, indicated that STCTs can boost adherence, clinical outcomes, and economic savings in most countries, but some considerations may be especially important in local markets. Much of the identified RWE and economic studies were based in the US and Europe. As a general recommendation, future research should include patients in other regions to shed light on global variation and common themes across STCT use and outcomes.

While this article may be especially useful for payers, the benefits of STCT use are relevant to multiple stakeholders, including patients, clinicians, and caregivers. For patients, improved treatment adherence with STCTs can result in positive clinical outcomes, reduced medical costs, and improved treatment satisfaction. STCT use helps to align patient actions with the recommendations of clinicians, contributing to positive clinical outcomes. Caregivers benefit from STCTs through reduced medication costs, co-pay costs, and drug administration burden. Finally, payers benefit from the reduced economic burden and HCRU that is accompanied by improved clinical outcomes.

### Strengths

The substantial amount of literature reporting outcomes of STCTs vs LDCs, spanning the last 2 decades and various countries, greatly contributed to the value of this study. The use of common measures in RWE studies and cost- effectiveness and budget impact analyses resulted in comparability across many studies. Furthermore, in contrast to previously published SLRs that primarily focused on the effects of STCTs in single indications including hypertension,^139,140^ diabetes,^16^ or HIV,^141^ our searches were not limited by indication or intervention, which allowed for broad commonalities to emerge in the comparison of STCTs and LDCs. Not only do the results of our SLR support the observations that use of STCTs may improve adherence and clinical effectiveness while also lowering costs as presented in previously published SLRs,^16,139–142^ our study further provides analysis among patients with Parkinson’s,^143^ asthma,^43^ and more general cardiovascular disease,^94,99,100^ which have previously not been reported.

### Limitations

Comparability in this study was limited by the variation in the outcomes that were measured and reported by the included studies. Additionally, in some cases, studies that did not report favorable results for the use of STCTs by one metric reported broadly favorable results for STCT use when considering other metrics. One example is a study that reported the results of switching from LDCs to STCTs in patients with type 2 diabetes mellitus.^58^ Although treatment adherence was high before and after switching, adherence slightly declined after patients switched to STCTs (92.4% to 90.9%).^58^ Despite this, hemoglobin A1C values were significantly improved with STCTs.^58^ The context of the original study, though largely favorable to STCT use, is lost when reporting aggregated adherence results.

The RWE findings clearly showed that STCT use is associated with improved outcomes but do not provide a mechanistic explanation for this within the context of drug administration. Since STCTs affect several distinct administration processes by reducing the number of medications to be prescribed, filled at the pharmacy, and consumed, studies that examine these processes directly could differentiate between benefits provided by STCTs and other administration options, like bundling prescriptions to be filled simultaneously at the pharmacy.^144^

Lastly, some heterogeneity in our results may be due to the inherent nature of different diseases that vary in patient population demographics, drug administration protocols, symptom severity, and perceived mortality risk—factors that can influence patient behavior and treatment adherence.

## CONCLUSIONS

The use of STCTs resulted in positive outcomes compared with LDCs across clinical studies, RWE, HRQoL studies, and economic evaluations. In RWE studies, STCTs were associated with consistent improvements in treatment adherence, persistence, and compliance, with evidence for greater adherence and persistence resulting in positive clinical outcomes. STCTs consistently dominated LDCs in cost-effectiveness and cost-utility analyses, and all identified budget impact analyses found cost savings with STCTs. This study provides a solid foundation for understanding the benefits of STCTs across clinical indications and the need for these to be thoughtfully considered in formulary decision-making. The findings highlight opportunities for future research to contribute by reporting STCT outcomes in additional countries and measuring drug administration–related HRQoL.

### Author Contributions

C.P. contributed to the planning, critical review of the manuscript, and approved the final manuscript; J.L. contributed to the planning, critical review of the manuscript, and approved the final manuscript; J.W. contributed to the planning, critical review of the manuscript, and approved the final manuscript; K.G. performed the systematic literature review, data extraction, and approved the final manuscript; D.T. performed the systematic literature review and data extraction, and approved the final manuscript; and S.G. performed the systematic literature review, data extraction, and approved the final manuscript.

### Disclosures

C.P. is an employee of Janssen Pharmaceutical Companies of Johnson & Johnson and J.L. is an employee of Janssen-Cliag of Johnson & Johnson, both with stock or stock options at Johnson & Johnson. J.W. was an employee of Janssen Pharmaceutical Companies of Johnson & Johnson at the time of the study. D.T. and S.G. are employees of Cytel, Inc. K.G. was an employee of Cytel, Inc. at the time of the study.

## Supplementary Material

Online Supplementary Material
