# Supplementary material for: Effectiveness of Single-Tablet Combination Therapy in Improving Adherence and Persistence and the Relation to Clinical and Economic Outcomes
Source: J Health Econ Outcomes Res. 2024 Jan 23;11(1):8–22. doi: 10.36469/001c.91396 (PMC10948140; doi:10.36469/001c.91396)
Supplement: Online Supplementary Material [file jheor_2024_11_1_91396_193089.pdf]

Journal of Health Economics and Outcomes Research

Online Supplementary Material

Effectiveness of Single-Tablet Combination Therapy in Improving Adherence and Persistence and the Relation to Clinical and Economic Outcomes. *JHEOR*. 2024;11(1):8-22. doi:10.36469/jheor.2024.91396

Table S1: Search Strategies **2**

Table S2: PICOS Criteria **11**

Table S3: Included Studies and Their Characteristics **13**

This supplementary material has been provided by the authors to give readers additional information about their work.

This is an open-access article distributed under the terms of the Creative Commons Attribution 4.0 International License (CCBY-4.0). View this license's legal deed at http://creativecommons.org/licenses/by/4.0 and legal code at http://creativecommons.org/licenses/by/4.0/legalcode for more information.

Supplementary Tables and Figures

Table S1. Search Strategies

**Search Strategy for RWE SLR**			
**Search conducted 3-Dec-21**			
**Databases searched**	EBM Reviews - Cochrane Database of Systematic Reviews <2005 to December 02, 2021>		
**Databases searched**	EBM Reviews - ACP Journal Club <1991 to November 2021>		
	EBM Reviews - Database of Abstracts of Reviews of Effects <1st Quarter 2016>		
	EBM Reviews - Cochrane Clinical Answers <November 2021>		
	EBM Reviews - Cochrane Central Register of Controlled Trials <October 2021>		
	EBM Reviews - Cochrane Methodology Register <3rd Quarter 2012>		
	EBM Reviews - Health Technology Assessment <4th Quarter 2016>		
	EBM Reviews - NHS Economic Evaluation Database <1st Quarter 2016>		
	Econlit <1886 to November 25, 2021>		
	Embase <1974 to 2021 December 02>		
	Ovid MEDLINE(R) ALL <1946 to December 02, 2021>		
		**Term**	**Hits**
**Fixed dose combination**	1	((fixed$ or single-pill or single pill) adj6 (combination$ or regimen$)).ti,ab.	31044
**Fixed dose combination Studies with FDC**	2	((drug$ or medication$) adj1 combin$).ti,ab.	55585
	3	(fixed-combination$ or polypill$ or combopill$ or multi-drug regimen$ or multidrug regimen$ or multi drug regimen$ or multiple drug regimen$ or single-pill-combination$).ti,ab.	13276
	**4**	**or/1-3**	**88020**
**Loose dose combination**	5	((free$ or loose$ or conventional or separate$ or two-pill$ or "two pill$" or 2-pill$ or "2 pill$" or three-pill$ or "three pill$" or 3-pill$ or "3 pill$" or multiple$ or dual$ or double$ or triple$) adj6 (combination$ or regimen$)).ti,ab.	133161
**Loose dose combination Studies with FDC and LDC**	6	(free-combination$ or loose-combination$).ti,ab.	1611
	7	(separate adj3 (agent$ or drug$ or medication$ or component$ or tablet$ or pill$)).ti,ab.	9133
	8	((dual or double or triple) adj3 therap$).ti,ab.	59871
	9	or/5-8	191786
	**10**	**4 and 9**	**9518**
**RWE studies**	11	exp case-control studies/ or exp case control study/	1473368
**ECON Outcomes**	12	exp cross-sectional studies/ or exp cross-sectional study/	853989
https://www.york.ac.uk/media/crd/Systematic_Reviews.pdf	13	exp cohort studies/ or exp cohort analysis/	3198576
	14	longitudinal studies/ or longitudinal study/	323752
	15	prospective studies/ or prospective study/	1433568
	16	retrospective studies/ or retrospective study/	2148875
	17	observational study/	369915
	18	follow-up studies/	2048320
	19	clinical study/	160991
	20	(case control or case-control).ti,ab.	328590
	21	(cohort adj1 (study or studies or analysis or analyses)).ti,ab.	701159
	22	((longitudinal or retrospective or prospective or cross sectional or cross-sectional) adj1 (study or studies or review or analysis or analyses or cohort$)).ti,ab.	2533425
	23	((follow up or follow-up or followup or observational or uncontrolled or non randomi#ed or nonrandomi#ed or non-randomi#ed or non-interventional or noninterventional or non interventional or pragmatic) adj1 (study or studies)).ti,ab.	517607
	24	(registry or register or database or claims or single center or single-	6297275

**Search Strategy for RWE SLR**			
		center or multicenter or multi-center or multi center or survey or record$ or chart review or real world or real-world).ti,ab.	
	25	follow up/	1768996
	26	cohort$.ti,ab.	1985360
	27	25 and 26	270083
	28	or/11-24,27	12272929
**Economic studies with FDC and LDC**	**29**	**10 and 28**	**2696**
**Irrelevant Study Design**	30	(addresses or bibliography or biography or case report or comment or congresses or consensus development conference or duplicate publication or editorial or guideline or in vitro or interview or lectures or letter or monograph or news or "newspaper article" or practice guideline or "review literature" or "review of reported cases" or review, academic or review, multicase or review, tutorial or twin study).pt.	4409495
	31	(animals/ not (humans/ and animals/)) or (animal/ not (human/ and animal/))	6065402
	32	case report/ or case reports/	4911406
	33	or/30-32	14769257
	34	29 not 33	2655
**Limits**	35	limit 34 to english language	2426
	36	limit 35 to human	2233
	37	limit 36 to yr="2001 -Current"	2002
**Final: RWE studies with FDC and LDC**	**38**	**remove duplicates from 36**	**1365**
	38	hospitalization/ or "length of stay"/ or Patient Admission/ or Child, Hospitalized/ or Adolescent, Hospitalized/ or child hospitalization/ or hospital patient/ or hospitalized adolescent/ or hospitalized child/ or hospital admission/	1181218
	39	(hospitali$ or length of stay$ or length of hospital stay$ or length of hospitali#ation$ or ICU or intensive care unit or inpatient or admission).tw.	1871768
	40	disease burden/	59364
	41	burden$.ti,ab.	701283
	42	(productivit$ or employ$).ti,ab.	1798111
	43	((work or working) adj1 (absen$ or loss$ or disabilit$ or abilit$ or impairment$ or limitation$ or incapacit$ or capacit$)).ti,ab.	37747
	44	(indirect?treatment?comparison$ or indirect?comparison$ or indirect treatment comparison$ or indirect comparison$).ti,ab.	7566
	45	(health technology assessment$ or health technolog$ or HTA).ti,ab.	28684
	46	or/11-45	8568453

**Search Strategy for Clinical SLR**	
**Search conducted**	3-Dec-21
**Databases searched**	EBM Reviews - Cochrane Database of Systematic Reviews <2005 to December 02, 2021>
	EBM Reviews - ACP Journal Club <1991 to November 2021>
	EBM Reviews - Database of Abstracts of Reviews of Effects <1st Quarter 2016>
	EBM Reviews - Cochrane Clinical Answers <November 2021>
	EBM Reviews - Cochrane Central Register of Controlled Trials <October 2021>
	EBM Reviews - Cochrane Methodology Register <3rd Quarter 2012>
	EBM Reviews - Health Technology Assessment <4th Quarter 2016>
	EBM Reviews - NHS Economic Evaluation Database <1st Quarter 2016>
	Econlit <1886 to November 25, 2021>
	Embase <1974 to 2021 December 02>
	Ovid MEDLINE(R) ALL <1946 to December 02, 2021>

Search Strategy for RWE SLR			
		**Term**	**Hits**
**Fixed dose combination**	1	((fixed$ or single-pill or single pill) adj6 (combination$ or regimen$)).ti,ab.	31044
	2	((drug$ or medication$) adj1 combin$).ti,ab.	55585
	3	(fixed-combination$ or polypill$ or combopill$ or multi-drug regimen$ or multidrug regimen$ or multi drug regimen$ or multiple drug regimen$ or single-pill-combination$).ti,ab.	13276
**Studies with FDC**	**4**	**or/1-3**	**88020**
**Loose dose combination**	5	((free$ or loose$ or conventional or separate$ or two-pill$ or "two pill$" or 2-pill$ or "2 pill$" or three-pill$ or "three pill$" or 3-pill$ or "3 pill$" or multiple$ or dual$ or double$ or triple$) adj6 (combination$ or regimen$)).ti,ab.	133161
	6	(free-combination$ or loose-combination$).ti,ab.	1611
	7	(separate adj3 (agent$ or drug$ or medication$ or component$ or tablet$ or pill$)).ti,ab.	9133
	8	((dual or double or triple) adj3 therap$).ti,ab.	59871
	9	or/5-8	191786
**Studies with FDC and LDC**	**10**	**4 and 9**	**9518**
**RCTs**	11	exp Randomized Controlled Trial/ or exp Random Allocation/ or exp randomization/	1420224
https://www.nice.org.uk/guidance/ng50/documents/search-strategies	12	exp Placebos/	437283
	13	exp Double-Blind Method/ or exp Single-Blind Method/	598217
	14	exp clinical trial/ or exp clinical trial, phase ii/ or exp clinical trial, phase iii/ or exp controlled clinical trial/	2569397
	15	exp controlled clinical trials as topic/ or exp Randomized Controlled Trials as Topic/ or exp clinical trials as topic/	784307
	16	exp Multicenter Study/	617015
	17	exp Randomized Controlled Trial/ or exp Random Allocation/ or exp randomization/	1420224
	18	exp placebo/	374039
	19	exp double blind procedure/ or exp single blind procedure/ or exp crossover procedure/	275829
	20	exp clinical trial/ or exp phase 2 clinical trial/ or exp phase 3 clinical trial/ or exp controlled clinical trial/	2569397
	21	exp "controlled clinical trial (topic)"/ or exp "clinical trial (topic)"/ or exp "randomized controlled trial (topic)"/	374353
	22	exp multicenter Study/	617015
	23	randomized controlled trial.pt.	1089538
	24	controlled clinical trial.pt.	187182
	25	random$.ti,ab,kw.	4223615
	26	blind$.ti,ab,kw.	1162771
	27	(placebo$ or assign* or allocat* or volunteer*).ti,ab,kw.	2670083
	28	(parallel$ or factorial$ or crossover* or cross over*).ti,ab,kw.	1180257
	29	trial.ti.	966420
	30	('phase 3' or 'phase 2' or 'phase III' or 'phase II').af.	598190

Search Strategy for RWE SLR			
	31	((single or double or triple) adj3 (blind* or mask* or dummy)).af.	1015034
	32	('double-blind' or 'double-blinded').af.	850357
	33	(open label or open-label).af.	221241
**SLR**	34	exp Meta-Analysis/ or exp Meta-Analysis as Topic/ or exp "Systematic Review"/	741228
https://www.nice.org.uk/guidance/ng50/documents/search-strategies	35	exp meta analysis/ or exp "meta analysis (topic)"/ or exp "systematic review"/	721760
	36	(meta analy* or meta-analy* or metanaly* or metaanaly*).ti,ab.	543491
	37	((systematic* or evidence*) adj3 (review* or overview*)).ti,ab.	682578
	38	(reference list* or bibliograph* or hand search* or manual search* or relevant journal*).ab.	131862
	39	(search strategy or search criteria or systematic search or study selection or data extraction).ab.	158607
	40	(search* adj4 literature).ab.	186482
	41	(medline or pubmed or cochrane or embase or psychlit or psyclit or psychinfo or psycinfo or cinahl or science citation index or bids or cancerlit).ab.	683065
	42	cochrane.jw.	54428
	43	((multiple treatment* or indirect or mixed) adj2 comparison*).ti,ab.	10588
	44	or/11-43	9863992
**RCTs or SLRs with FDC and LDC**	**45**	**10 and 44**	**4885**
**Irrelevant Study Design**	46	(addresses or bibliography or biography or case report or comment or congresses or consensus development conference or duplicate publication or editorial or guideline or in vitro or interview or lectures or letter or monograph or news or "newspaper article" or practice guideline or "review literature" or "review of reported cases" or review, academic or review, multicase or review, tutorial or twin study).pt.	4409495
	47	(animals/ not (humans/ and animals/)) or (animal/ not (human/ and animal/))	6065402
	48	case report/ or case reports/	4911406
	49	or/46-48	14769257
	50	45 not 49	4780
**Limits**	51	limit 50 to english language	4177
	52	limit 51 to human	3916
	53	limit 52 to yr="2001 -Current"	3317
**Final: RCTs or SLRs with FDC and LDC**	**54**	**Deduplicate**	**2101**
**Search Strategy for HRQoL SLR**			
**Search conducted**	3-Dec-21		
**Databases**	EBM Reviews - Cochrane Database of Systematic Reviews <2005 to December 02, 2021>		

**Search Strategy for RWE SLR**			
**searched**	EBM Reviews - ACP Journal Club <1991 to November 2021>		
	EBM Reviews - Database of Abstracts of Reviews of Effects <1st Quarter 2016>		
	EBM Reviews - Cochrane Clinical Answers <November 2021>		
	EBM Reviews - Cochrane Central Register of Controlled Trials <October 2021>		
	EBM Reviews - Cochrane Methodology Register <3rd Quarter 2012>		
	EBM Reviews - Health Technology Assessment <4th Quarter 2016>		
	EBM Reviews - NHS Economic Evaluation Database <1st Quarter 2016>		
	Econlit <1886 to November 25, 2021>		
	Embase <1974 to 2021 December 02>		
	Ovid MEDLINE(R) ALL <1946 to December 02, 2021>		
		**Term**	**Hits**
**Fixed dose combination**	1	((fixed$ or single-pill or single pill) adj6 (combination$ or regimen$)).ti,ab.	31044
	2	((drug$ or medication$) adj1 combin$).ti,ab.	55585
	3	(fixed-combination$ or polypill$ or combopill$ or multi-drug regimen$ or multidrug regimen$ or multi drug regimen$ or multiple drug regimen$ or single-pill-combination$).ti,ab.	13276
**Studies with FDC**	**4**	**or/1-3**	**88020**
**Loose dose combination**	5	((free$ or loose$ or conventional or separate$ or two-pill$ or "two pill$" or 2-pill$ or "2 pill$" or three-pill$ or "three pill$" or 3-pill$ or "3 pill$" or multiple$ or dual$ or double$ or triple$) adj6 (combination$ or regimen$)).ti,ab.	133161
	6	(free-combination$ or loose-combination$).ti,ab.	1611
	7	(separate adj3 (agent$ or drug$ or medication$ or component$ or tablet$ or pill$)).ti,ab.	9133
	8	((dual or double or triple) adj3 therap$).ti,ab.	59871
	9	or/5-8	191786
**Studies with FDC and LDC**	**10**	**4 and 9**	**9518**
**QOL Outcomes**	11	exp "Quality of Life"/ or exp "Surveys and Questionnaires"/ or exp questionnaire/ or exp "quality of life assessment"/	2590945
http://nicedsu.org.uk/wp-content/uploads/2016/03/TSD9-HSUV-values_FINAL.pdf	12	(QOL$ or HQL$ or HQOL$ or H QOL$ or HRQL$ or HRQOL$ or HR QOL$).tw.	215343
	13	(quality adj4 life).tw.	987285
	14	(quality adj2 well?being).tw.	1243
	15	Quality-Adjusted Life Years/ or quality adjusted life year/	48994
	16	(quality adjusted life$ or quality-adjusted life$ or quality-adjusted-life$ or disability adjusted life$ or disability-adjusted life$ or disability-adjusted-life$).tw.	57604
	17	(QALY or qal$ or qwb$ or qald$ or qale$ or qtime$ or daly$).tw.	54242
	18	Patient Reported Outcome Measures/ or patient-reported outcome/	46286
	19	(patient adj2 reported adj2 outcome$).tw.	79069
	20	PRO.tw.	570637
	21	exp Health Status/ or exp Health Surveys/ or exp health survey/	1466999

Search Strategy for RWE SLR			
	22	(euroqol$ or euro qol$ or euro-qol$ or euroqual$ or euro qual$ or euro-qual$ or eq5d$ or eq 5d$ or eq-5d$ or eqoL-5d$ or eqoL5D$ or eqoL 5d$).tw.	51388
	23	(utilit$ or disutilit$).tw.	618610
	24	(hye$ or health$ year$ equivalent$ or hui$).tw.	15809
	25	(standard gamble$ or time-trade-off or time trade-off or time trade off or time tradeoff or discrete choice experiment$ or rosser).tw.	12030
	26	(willingness adj4 pay).tw.	26583
	27	(SG or TTO or WTP or DCE).tw.	59159
	28	((valu$ or measur$ or preference$) adj4 (health or outcome$ or effect$ or [illegible]	1619277
	29	(VAS or visual analog$ scale$ or visual-analog$ scale$).tw.	280328
	30	(sf-36$ or sf36$ or sf 36$ or sf thirtysix or sfthirtysix or sf-thirtysix or sf thirty six or sf-20$ or sf20$ or sf 20$ or sf twenty or sftwenty or sf-twenty or sf-12$ or sf12$ or sf 12$ or sf twelve or sftwelve or sf-twelve or sf-6$ or sf6$ or sf 6$ or sf six$ or sfsix$ or sf-six$ or short form$ or shortform$ or RAND$).tw.	4305530
	31	(satisf$ or Treatment Satisfaction Questionnaire or TSQM$).tw.	957068
	32	patient compliance/ or medication adherence/	255854
	33	(adhere$ or complian$ or persist$ or cooperation or coperation or co-operation or comply or discontinu$ or non-compliance or non compliance or noncompliance or non-adherence or nonadherence or non adherence or non persisten$ or nonpersisten$ or non-persisten$).tw.	2752430
	34	or/11-33	12027593
**QOL studies with FDC and LDC**	**35**	**10 and 34**	**4852**
**Irrelevant Study Design**	36	(addresses or bibliography or biography or case report or comment or congresses or consensus development conference or duplicate publication or editorial or guideline or in vitro or interview or lectures or letter or monograph or news or "newspaper article" or practice guideline or "review literature" or "review of reported cases" or review, academic or review, multicase or review, tutorial or twin study).pt.	4409495
	37	(animals/ not (humans/ and animals/)) or (animal/ not (human/ and animal/))	6065402
	38	case report/ or case reports/	4911406
	39	or/36-38	14769257
	40	35 not 39	4729
**Limits**	41	limit 40 to english language	4159
	42	limit 41 to human	3830
	43	limit 42 to yr="2001 -Current"	3345
**Final: QOL studies with FDC and LDC**	**44**	**Deduplicate**	**2073**
**Search Strategy for Economic SLR**			
**Search conducted**	3-Dec-21		
**Databases searched**	EBM Reviews - Cochrane Database of Systematic Reviews <2005 to December 02, 2021>		
	EBM Reviews - ACP Journal Club <1991 to November 2021>		
	EBM Reviews - Database of Abstracts of Reviews of Effects <1st Quarter 2016>		
	EBM Reviews - Cochrane Clinical Answers <November 2021>		
	EBM Reviews - Cochrane Central Register of Controlled Trials <October 2021>		
	EBM Reviews - Cochrane Methodology Register <3rd Quarter 2012>		
	EBM Reviews - Health Technology Assessment <4th Quarter 2016>		
	EBM Reviews - NHS Economic Evaluation Database <1st Quarter 2016>		
	Econlit <1886 to November 25, 2021>		

Search Strategy for RWE SLR			
	Embase <1974 to 2021 December 02>		
	Ovid MEDLINE(R) ALL <1946 to December 02, 2021>		
		**Term**	**Hits**
**Fixed dose combination**	1	((fixed$ or single-pill or single pill) adj6 (combination$ or regimen$)).ti,ab.	31044
	2	((drug$ or medication$) adj1 combin$).ti,ab.	55585
	3	(fixed-combination$ or polypill$ or combopill$ or multi-drug regimen$ or multidrug regimen$ or multi drug regimen$ or multiple drug regimen$ or single-pill-combination$).ti,ab.	13276
**Studies with FDC**	**4**	**or/1-3**	**88020**
**Loose dose combination**	5	((free$ or loose$ or conventional or separate$ or two-pill$ or "two pill$" or 2-pill$ or "2 pill$" or three-pill$ or "three pill$" or 3-pill$ or "3 pill$" or multiple$ or dual$ or double$ or triple$) adj6 (combination$ or regimen$)).ti,ab.	133161
	6	(free-combination$ or loose-combination$).ti,ab.	1611
	7	(separate adj3 (agent$ or drug$ or medication$ or component$ or tablet$ or pill$)).ti,ab.	9133
	8	((dual or double or triple) adj3 therap$).ti,ab.	59871
	9	or/5-8	191786
**Studies with FDC and LDC**	**10**	**4 and 9**	**9518**
**ECON Outcomes**	11	exp "economic evaluation"/	413888
https://www.york.ac.uk/media/crd/Systematic_Reviews.pdf	12	economics/ or economic aspect/	380367
	13	Economics, Pharmaceutical/ or health economics/ or pharmacoeconomics/	45621
	14	cost-benefit analysis/ or "cost effectiveness analysis"/ or "cost minimization analysis"/ or "cost benefit analysis"/ or "cost utility analysis"/	403275
	15	((economic or human$) adj3 consequence$).ti,ab	21174
	16	(economic$ or pharmaco?economic$ or pharmaco economic$).ti,ab	1074840
	17	(cost$ adj2 (effective$ or utilit$ or benefit$ or minimi$ or consequence$)).ti,ab	487682
	18	(CEA or CMA or CBA or CUA or CCA).ti,ab.	125486
	19	models, economic/ or economic model/	14993
	20	decision theory/ or decision trees/ or "decision tree"/	31553
	21	monte carlo method/	75798
	22	(econom$ model$).ti,ab.	27158
	23	markov$.ti,ab	72387
	24	(discrete-event simulation$ or discrete event simulation$ or [illegible]	7384
	25	"monte carlo".ti,ab	118267
	26	(decision$ adj2 (tree$ or anal$ or model$)).ti,ab	76602
	27	("de novo" adj1 model$).ti,ab	446
	28	budgets/ or budget/	42654
	29	budget$.ti,ab.	100828
	30	"Costs and Cost Analysis"/ or cost/	114281

Search Strategy for RWE SLR			
	31	"cost of illness"/	51766
	32	health care costs/ or "health care cost"/ or health expenditures/	271255
	33	cost$.ti,ab.	1849786
	34	Drug Utilization/ or "drug use"/	165140
	35	Health Resources/ or health care utilization/	239672
	36	((resource$ or health care or healthcare or health service$ or drug$ or medication$) adj4 (use$ or usage$ or utilit$ or utili#ation$)).ti,ab	969441
	37	"hospitalization cost"/	8216
	38	hospitalization/ or "length of stay"/ or Patient Admission/ or Child, Hospitalized/ or Adolescent, Hospitalized/ or child hospitalization/ or hospital patient/ or hospitalized adolescent/ or hospitalized child/ or hospital admission/	1181218
	39	(hospitali$ or length of stay$ or length of hospital stay$ or length of hospitali#ation$ or ICU or intensive care unit or inpatient or admission).tw.	1871768
	40	disease burden/	59364
	41	burden$.ti,ab.	701283
	42	(productivit$ or employ$).ti,ab.	1798111
	43	((work or working) adj1 (absen$ or loss$ or disabilit$ or abilit$ or impairment$ or limitation$ or incapacit$ or capacit$)).ti,ab.	37747
	44	(indirect?treatment?comparison$ or indirect?comparison$ or indirect treatment comparison$ or indirect comparison$).ti,ab.	7566
	45	(health technology assessment$ or health technolog$ or HTA).ti,ab.	28684
	46	or/11-45	8568453
**Economic studies with FDC and LDC**	**47**	**10 and 46**	**2626**
**Irrelevant Study Design**	48	(addresses or bibliography or biography or case report or comment or congresses or consensus development conference or duplicate publication or editorial or guideline or in vitro or interview or lectures or letter or monograph or news or "newspaper article" or practice guideline or "review literature" or "review of reported cases" or review, academic or review, multicase or review, tutorial or twin study).pt.	4409495
	49	(animals/ not (humans/ and animals/)) or (animal/ not (human/ and animal/))	6065402
	50	case report/ or case reports/	4911406
	51	or/48-50	14769257
	52	47 not 51	2480
**Limits**	53	limit 52 to english language	2287
	54	limit 53 to human	1945
	55	limit 54 to yr="2001 -Current"	1702
**Final: Economic studies with FDC and LDC**	**56**	**Deduplicate**	**1145**

**Search Strategy for RWE SLR**	
**Conference Search**	
**Date searched**	**27th January 2022**
**Keywords used**	**Fixed dose combination**
**No. of hits**	**26**

Abbreviations: ECON, economic; FDC, fixed dose combination; HRQoL, health-related quality of life; LDC, loose dose combination; QOL, quality of life; RCT, randomized clinical trial; RWE, real-world evidence; SLR, systematic literature review

Table S2. PICOS Criteria

**RWE**	
**Population**	Any population
**Interventions**	Fixed dose combinations
**Comparator**	Loose combination products
**Outcomes**	Any efficacy outcomes
	Any safety outcomes
	Adherence, persistence, compliance-related outcomes
**Study Design**	Real-world (observational) studies
	SLRs and meta-analyses (for cross-checking only)
**Other Limits**	Time: 2001 to current
	English language
	Studies conducted in the US, Canada, EU, Australia
**Clinical Trials**	
**Population**	Patients with chronic conditions
**Interventions**	Fixed dose combinations
**Comparator**	Loose combination products
**Outcomes**	Any efficacy outcomes
	Any safety outcomes
	Any patient-reported outcomes
	Adherence, persistence, compliance-related outcomes
**Study Design**	Interventional (RCTs, clinical trials)
	SLRs and meta-analyses (for cross-checking only)
**Other Limits**	Time: 2001 to current
	English language
**HRQoL Studies**	
**Population**	Any population
**Interventions**	Fixed dose combinations
**Comparator**	Loose combination products
**Outcomes**	Any health-related quality of life measures
	Any patient-reported outcome measures
	Symptoms
	Patient preferences
**Study Design**	RWE or RCTs assessing HRQoL/PROs
	SLRs and meta-analyses (for cross-checking only)
**Other Limits**	Time: 2001 to current

	English language
**Economic Studies**	
**Population**	Any population
**Interventions**	Fixed dose combinations
**Comparator**	Loose combination products
**Outcomes**	Direct medical costs
	Indirect medical costs
	Healthcare resource use and cost parameters
	ICERs, QALYs
	Utilities
	Costs related to budget impact
**Study Design**	Interventional, real-world studies, and economic evaluations assessing cost/HCRU
	SLRs and meta-analyses (for cross-checking only)
**Other Limits**	Time: 2001 to current
	English language

Abbreviations: EU, European Union; HCRU, healthcare resource utilization; ICER, incremental cost-effectiveness ratio; PICOS, population, intervention, comparator, outcomes, and study; PRO, patient-reported outcome; QALY, quality-adjusted life year; RCT, randomized clinical trial; RWE, real-world evidence; SLR, systematic literature review;.

Table S3. Included Studies and Their Characteristics

Citation	SLR	Indication	Study Design
Ambery P, Stylianou A, Atkinson G, et al. Open-label randomized non-inferiority trial of a fixed-dose combination of glimepiride and atorvastatin for the treatment of people whose Type 2 diabetes is uncontrolled on metformin. *Diabet Med.* 2016;33(8):1084-1093. https://dx.doi.org/10.1111/dme.13003 [70]	Clinical	Type 2 diabetes mellitus	Phase 3, RCT, open-label, parallel group, multicenter
Aseffa A, Chukwu JN, Vahedi M, et al. Efficacy and safety of 'fixed dose' versus 'loose' drug regimens for treatment of pulmonary tuberculosis in two high TB-burden African countries: a randomized controlled trial. *PloS One*. 2016;11(6):e0157434. doi:10.1371/journal.pone.0157434 [57]	Clinical	Tuberculosis	Phase not reported, RCT, single-blind, non-inferiority, multicenter
Ashley EA, Lwin KM, McGready R, et al. An open label randomized comparison of mefloquine-artesunate as separate tablets vs. a new co-formulated combination for the treatment of uncomplicated multidrug-resistant falciparum malaria in Thailand. *Trop Med Int Health*. 2006;11(11):1653-1660.[109]	Clinical	Symptomatic falciparum or mixed malarial infection	Phase not reported, RCT, open-label
Barnebey HS, Robin AL. Adherence to fixed-combination versus unfixed travoprost 0.004%/timolol 0.5% for glaucoma or ocular hypertension: a randomized trial. *Am J Ophthalmol*. 2017;176:61-69. doi:10.1016/j.ajo.2016.12.002[69]	Clinical	Open-angle glaucoma or ocular hypertension	Phase 3, RCT, observer-blind, multicenter
Barner JC. Adherence to oral antidiabetic agents with pioglitazone and metformin: comparison of fixed-dose combination therapy with monotherapy and loose-dose combination therapy. *Clin Ther*. 2011;33(9):1281-1288. doi:10.1016/j.clinthera.2011.07.016 [21]	RWE	Type 2 diabetes mellitus	Retrospective, database analysis
Bartacek A, Schütt D, Panosch B, et al. Comparison of a four-drug fixed-dose combination regimen with a single tablet regimen in smear-positive pulmonary tuberculosis. *Int J Tuberc Lung Dis*. 2009;13(6):760-766. [110]	Clinical	Tuberculosis	Phase not reported, RCT, open-label, multicenter
Beck EJ, Mandalia S, Sangha R, et al. Lower healthcare costs associated with the use of a single-pill ARV regimen in the UK, 2004–2008. *PLoS One*. 2012;7(10):e47376. doi:10.1371/journal.pone.0047376 [93]	Economic, RWE	HIV	Retrospective, database analysis
Belfort R, Gabriel L, Martins Bispo PJ, et al. Safety and efficacy of moxifloxacin-dexamethasone eyedrops as treatment for bacterial ocular infection associated with bacterial blepharitis. *Adv Ther*. 2012;29(5):416-426. doi:10.1007/s12325-012-0018-8 [111]	Clinical, HRQoL	Bacterial blepharitis and/or keratitis and/or conjunctivitis with or without corneal inflammation	Phase not reported, RCT, double-blind, active-controlled, parallel-group
Bhagat P, Sodimalla K, Paul C, et al. Efficacy and safety of benzalkonium chloride-free fixed-dose combination of latanoprost and timolol in patients with open-angle glaucoma or ocular hypertension. *Clin Ophthalmol*. 2014;8:1241-1252. doi:10.2147/OPTH.S64584 [112]	Clinical	Open-angle glaucoma or ocular hypertension	Phase 3, RCT, open-label, parallel-group, active-controlled, multicenter
Blonde L, Wogen J, Kreilick C, Seymour AA. Greater reductions in A1C in type 2 diabetic patients new to therapy with glyburide/metformin tablets as compared to glyburide co-administered with metformin. doi:10.1046/j.1463-1326.2003.00297.x. *Diabet Obes Metab*. 2003/11/01 2003;5(6):424-431. doi:10.1046/j.1463-1326.2003.00297.x[22]	RWE	Type 2 diabetes mellitus	Retrospective, database analysis
Bodzenta-Lukaszyk A, Pulka G, Dymek A, et al. Efficacy and safety of fluticasone and formoterol in a single pressurized metered dose inhaler. *Resp Med*. 2011;105(5):674-682. doi:10.1016/j.rmed.2010.11.011 [113]	Clinical, HRQoL	Asthma	Phase 3, RCT, double-blind, multicenter

Citation	SLR	Indication	Study Design
Bohm AK, Schneider U, Aberle J, Stargardt T. Regimen simplification and medication adherence: Fixed-dose versus loose-dose combination therapy for type 2 diabetes. *PLoS One*. 2021;16(5):e0250993. doi:10.1371/journal.pone.0250993 [23]	RWE	Type 2 diabetes mellitus	Retrospective, database analysis
Bosworth C, de Boer IH, Targher G, Kendrick J, Smits G, Chonchol M. The effect of combined calcium and cholecalciferol supplementation on bone mineral density in elderly women with moderate chronic kidney disease. *Clin Nephrol*. 2012;77(5):358-365. [114]	Clinical	Kidney disease	Phase 3, RCT, double-blind, comparative, placebo-controlled
Bramlage P, Schmidt S, Sims H. Fixed-dose vs free-dose combinations for the management of hypertension-an analysis of 81 958 patients. *J Clin Hypertens (Greenwich)*. 2018;20(4):705-715. doi:10.1111/jch.13240 [24]	Economic, RWE	Hypertension	Retrospective, database analysis
Bricout-Hennel S, Zelveian P, Nedogoda S. Safety and efficacy of indapamide sustained release/amlodipine fixed-dose combination in essential hypertension. Journal of Hypertension 2018;36(Supplement 1):e45. [115]	Clinical	Hypertension	Phase 3, RCT, open-label, multicenter
Brixner DI, Jackson IIKC, Sheng X, et al. Assessment of adherence, persistence, and costs among valsartan and hydrochlorothiazide retrospective cohorts in free-and fixed-dose combinations. Current Medical Research and Opinion 2008;24(9):2597-607. doi: 10.1185/03007990802319364 [25]	Economic, RWE	Hypertension	Retrospective, database analysis
Brüggenjürgen B, Ezzat N, Kardos P, et al. Economic evaluation of BDP/formoterol fixed vs two single inhalers in asthma treatment. Allergy 2010;65(9):1108-15. doi: https://doi.org/10.1111/j.1398-9995.2009.02317.x [84]	Economic	Asthma	Cost minimization analysis
Campos M, Muccioli C, Malta JBNS, et al. Efficacy and tolerability of a combined gatifloxacin plus prednisolone formulation for topical prophylaxis after LASIK. Clinical Ophthalmology 2011;5(1):209-14. doi: http://dx.doi.org/10.2147/OPTH.S17059 [78]	Clinical, HRQoL	Laser-assisted in situ keratomileusis (LASIK) recovery	Phase not reported, RCT, double-blind, parallel-group, comparative study
Castellano JM, Sanz G, Penalvo JL, et al. A polypill strategy to improve adherence: results from the FOCUS project. Journal of the American College of Cardiology 2014;64(20):2071-82. doi: https://dx.doi.org/10.1016/j.jacc.2014.08.021 [66]	Clinical	Acute myocardial infarction	Phase 2, RCT, open-label, active-controlled, piggyback, 2-group parallel, multicenter
Chen W, Wei Z, Ong SH, et al. Health Care Utilization and Cost Comparison Between Adherent Hypertension Patients Treated by Single Exforge HCT and Amlodipine/Valsartan/Hydrochlorothiazide Free Combination. Value in Health 2014;17(7):A723. doi: 10.1016/j.jval.2014.08.036 [94]	Economic	Hypertension	Retrospective, cohort
Cheong C, Barner JC, Lawson KA, et al. Patient adherence and reimbursement amount for antidiabetic fixed-dose combination products compared with dual therapy among texas medicaid recipients. Clinical Therapeutics 2008;30(10):1893-907. doi: 10.1016/j.clinthera.2008.10.003 [26]	Economic, RWE	Diabetes	Retrospective, database analysis
Cohen CJ, Kubota M, Brachman PS, et al. Short-term safety and tolerability of a once-daily fixed-dose abacavir-lamivudine combination versus twice-daily dosing of abacavir and lamivudine as separate components: findings from the ALOHA study. Pharmacotherapy 2008;28(3):314-22. doi: https://dx.doi.org/10.1592/phco.28.3.314 [58]	Clinical, HRQoL	HIV	Phase 3b, RCT, open-label, parallel-group, multicenter
Czarnecka D, Koch EMW, Gottwald-Hostalek U. Benefits of a fixed-dose combination of bisoprolol and amlodipine in the treatment of hypertension in daily practice: results of more than 4000 patients.	RWE	Hypertension	Prospective, Multicenter

Citation	SLR	Indication	Study Design
Current Medical Research and Opinion 2015;31(5):875-81. doi: 10.1185/03007995.2015.1027676 [38]			
da Cunha PAF, Shinzato FA, Tecchio GT, et al. Efficacy and tolerability of a gatifloxacin/prednisolone acetate fixed combination for topical prophylaxis and control of inflammation in phacoemulsification: a 20-day-double-blind comparison to its individual components. Clinics 2013;68(6):834-39. doi: https://doi.org/10.6061/clinics/2013(06)18 [116]	Clinical	Cataract	Phase not reported, RCT, double-blind, parallel-group
Dahl R, Jadayel D, Alagappan V, et al. Efficacy and safety of once-daily QVA149 compared with the free combination of its monocomponents: The beacon study. Chest 2014;145(3 MEETING ABSTRACT) doi: http://dx.doi.org/10.1378/chest.1824459 [117]	Clinical, HRQoL	COPD	Phase 3, RCT, double-blind, parallel group, non-inferiority, multicenter
Dahl R, Jadayel D, Alagappan VKT, et al. Efficacy and safety of QVA149 compared to the concurrent administration of its monocomponents indacaterol and glycopyrronium: the BEACON study. International journal of chronic obstructive pulmonary disease 2013;8:501-8. doi: https://dx.doi.org/10.2147/COPD.S49615 [79]	Clinical, HRQoL	COPD	Phase 3, RCT, double-blind, parallel group, non-inferiority, multicenter
Dahl R, Jadayel D, Alagappan V, Chen H, Banerji D. Once-daily QVA149 provides the same efficacy as the free combination of its monocomponents indacaterol and glycopyrronium: The BEACON study. European respiratory journal, 42 (2013). [118]	Clinical, HRQoL	COPD	Phase 3, RCT, double-blind, parallel group, non-inferiority, multicenter
Degli Esposti L, Perrone V, Veronesi C, et al. Modifications in drug adherence after switch to fixed-dose combination of perindopril/amlodipine in clinical practice. Results of a large-scale Italian experience. The amlodipine-perindopril in real settings (AMPERES) study. Current Medical Research and Opinion 2018;34(9):1571-77. doi: 10.1080/03007995.2018.1433648 [27]	RWE	Hypertension	Retrospective, database analysis
Delea TE, Arondekar B, Kartashov A. Add-on Therapy with Rosiglitazone (RSG)/Metformin (MET) as a Fixed-Dose Combination (FDC) vs RSG plus MET or RSG plus Sulfonylurea (SU) as Separate Pills (SP): Retrospective Study of Outcomes and Costs. Diabetes 2007;56:2200-P0. [36]	Economic, RWE	Type 2 diabetes mellitus	Retrospective, database analysis
Delea TE, Thomas SK, Hagiwara M, et al. Adherence with levodopa/carbidopa/entacapone versus levodopa/carbidopa and entacapone as separate tablets in patients with Parkinson's disease. Current Medical Research and Opinion 2010;26(7):1543-52. doi: 10.1185/03007991003780628 [40]	RWE	Parkinson disease	Retrospective, database analysis
Diestelhorst M, Larsson L, European-Canadian Latanoprost Fixed Combination Study Group. A 12-week, randomized, double-masked, multicenter study of the fixed combination of latanoprost and timolol in the evening versus the individual components. Ophthalmology, 113(1), 70 (2006). [119]	Clinical	Ocular hypertension or primary open-angle, pigmentary or exfoliative glaucoma	Phase not reported, RCT, double-blind, multicenter
Duckworth W. Improvements in Glycemic Control in Type 2 Diabetes Patients Switched From Sulfonylurea Coadministered With Metformin to Glyburide-Metformin Tablets. Journal of Managed Care Pharmacy 2003;9(3):256-62. doi: 10.18553/jmcp.2003.9.3.256 [37]	RWE	Type 2 diabetes mellitus	Retrospective, database analysis
Ehlken B, Kostev K, Breitscheidel L, et al. PCV104 Persistence in Hypertension Treatment with Olmesartan Medoxomil Versus Valsartan - Analysis of Real-Life Prescription Data in Germany. Value in Health 2011;14(7):A383. doi: 10.1016/j.jval.2011.08.824	RWE	Hypertension	Retrospective, database analysis
Finelli R, Pascale AV, Battimelli A, et al. Calcium channel blocker and angiotensin-converting enzyme (ACE) inhibitor antihypertensive	Clinical	Hypertension	Phase not reported, RCT

Citation	SLR	Indication	Study Design
regimen: Comparing fixed vs. Free combination therapy. High Blood Pressure and Cardiovascular Prevention 2014;21(4):333-34. doi: http://dx.doi.org/10.1007/s40292-014-0066-z [120]			
Francis BA, Du LT, Berke S, et al. Comparing the fixed combination dorzolamide-timolol (Cosopt) to concomitant administration of 2% dorzolamide (Trusopt) and 0.5% timolol -- a randomized controlled trial and a replacement study. Journal of clinical pharmacy and therapeutics 2004;29(4):375-80. [121]	Clinical	Open-angle glaucoma, ocular hypertension or pseudo exfoliation glaucoma	Phase not reported, RCT, multicenter
Fujii RK, Restrepo M, Fernandes RA, et al. Cost-Effectiveness Analysis And Budget Impact Of Concor® Am Versus Bisoprolol Plus Amlodipine In Systemic Arterial Hypertension Treatment, From The Perspective Of The Brazilian Public Health System. Value in Health 2015;18(3):A138. doi: 10.1016/j.jval.2015.03.801 [85]	Economic	Hypertension	CEA, BIA
Gaciong Z, Hostalek U, Kurzeja A. Compliance and acceptance of fixed-dose combination of bisoprolol and aspirin. Open-label multicenter study. Journal of Hypertension, 35, e340-e341 (2017). [56]	RWE	Hypertension and/or coronary heart disease	Prospective, Multicenter
Glezer M. Approaches to increase efficacy of antihypertensive treatment: Results of the Russian observational program forsage. Journal of Hypertension 2016;34(Supplement 2):e297-e98. doi: http://dx.doi.org/10.1097/01.hjh.0000523870.41484.74 [73]	HRQoL	Hypertension	Prospective, observational, multicenter
Hagedorn C, Kässner F, Banik N, et al. Influence of salmeterol/fluticasone via single versus separate inhalers on exacerbations in severe/very severe COPD. Respiratory Medicine 2013;107(4):542-49. doi: 10.1016/j.rmed.2012.12.020 [42]	HRQoL, Economic, RWE	COPD	Prospective, multicenter
Hollo G, Hommer A, Anton A, et al. Preservative-free tafluprost 00015%/timolol 05% fixed dose combination: A 6-month double-masked, randomized multicenter P-III comparison to concomitant use of the individual preservative-free components in patients with glaucoma or ocular hypertension. 2013 Annual Meeting of the Association for Research in Vision and Ophthalmology 2014;54(15) [122]	Clinical, HRQoL	Ocular hypertension or open-angle glaucoma	Phase 3, RCT, double-blind, active-controlled, parallel-group, multicenter, multinational
Hollo G, Hommer A, Anton Lopez A, et al. Efficacy, safety, and tolerability of preservative-free fixed combination of tafluprost 0.0015%/timolol 0.5% versus concomitant use of the ingredients. Journal of ocular pharmacology and therapeutics : the official journal of the Association for Ocular Pharmacology and Therapeutics 2014;30(6):468-75. doi: https://dx.doi.org/10.1089/jop.2013.0229 [123]	Clinical, HRQoL	Ocular hypertension or open-angle glaucoma	Phase 3, RCT, double-blind, active-controlled, parallel-group, multicenter, multinational
Hollo G, Ropo A. Intraocular pressure decrease with preservative-free fixed and unfixed combination of tafluprost and timolol in pseudoexfoliative glaucoma. Current medical research and opinion 2015;31(1):13-6. doi: https://dx.doi.org/10.1185/03007995.2014.972500 [80]	Clinical, HRQoL	Ocular hypertension or open-angle glaucoma	Phase 3, RCT, double-blind, active-controlled, parallel-group, multicenter, multinational
Hommer A GIGI. A double-masked, randomized, parallel comparison of a fixed combination of bimatoprost 0.03%/timolol 0.5% with non-fixed combination use in patients with glaucoma or ocular hypertension. European journal of ophthalmology 2007;17(1):53. [124]	Clinical	Ocular hypertension or glaucomatous disease	Phase 3, RCT, double-blind, parallel groups
Hong SH, Wang J, Tang J. Dynamic View on Affordability of Fixed-Dose Combination Antihypertensive Drug Therapy. American Journal of Hypertension 2013;26(7):879-87. doi: 10.1093/ajh/hpt035 [95]	Economic	Hypertension	Retrospective database analysis
Hostalek U, Czarnecka D, Koch EMW. Treatment of Hypertensive Patients with a Fixed-Dose Combination of Bisoprolol and	RWE	Hypertension	Prospective, multicenter

Citation	SLR	Indication	Study Design
Amlodipine: Results of a Cohort study with More Than 10,000 Patients. Cardiology and Therapy 2015;4(2):179-90. doi: 10.1007/s40119-015-0045-z [41]			
Huchon G, Magnussen H, Chuchalin A, et al. Lung function and asthma control with beclomethasone and formoterol in a single inhaler. Respiratory medicine 2009;103(1):41-9. doi: https://dx.doi.org/10.1016/j.rmed.2008.09.002 [125]	Clinical	Asthma	Phase 3, RCT, double-blind, double-dummy, multicenter
Inoue K, Shiokawa M, Sugahara M, et al. Three-month evaluation of dorzolamide hydrochloride/timolol maleate fixed-combination eye drops versus the separate use of both drugs. Japanese journal of ophthalmology 2012;56(6):559-63. doi: https://dx.doi.org/10.1007/s10384-012-0186-8 [126]	HRQoL	Open-angle glaucoma or ocular hypertension	Prospective, cohort, multicenter
Jackson K, Brixner D, Oderda G, et al. Compliance and persistence of fixed dose versus free dose combination therapy with valsartan and HCTZ for patients with hypertension. Value in Health 2006;9 doi: 10.1016/S1098-3015(10)63700-X [52]	RWE	Hypertension	Retrospective, database analysis
Jenkins C, Kolarikova R, Kuna P, et al. Efficacy and safety of high-dose budesonide/formoterol (Symbicort®) compared with budesonide administered either concomitantly with formoterol or alone in patients with persistent symptomatic asthma. Respirology 2006;11(3):276-86. doi: https://doi.org/10.1111/j.1440-1843.2006.00856.x [59]	Clinical, HRQoL	Asthma	Phase not reported, RCT, double-blind, double-dummy
Kamat SA, Bullano MF, Chang C-L, et al. Adherence to single-pill combination versus multiple-pill combination lipid-modifying therapy among patients with mixed dyslipidemia in a managed care population. Current Medical Research and Opinion 2011;27(5):961-68. doi: 10.1185/03007995.2011.562494 [28]	RWE	Mixed dyslipidemia	Retrospective, database analysis
Kawalec P, Holko P, Stawowczyk E, et al. Economic evaluation of single-pill combination of indapamide and amlodipine in the treatment of arterial hypertension in the Polish setting. Kardiologia Polska (Polish Heart Journal) 2015;73(9):768-80. doi: 10.5603/KP.a2015.0089 [86]	Economic	Hypertension	CEA/CUA
Kawalec P, Stawowczyk E, Holko P, et al. Budget Impact Analysis Of Hypertensive Treatment With Indapamide And Amlodipine Single-Pill Combination In The Polish Setting. Value in Health 2014;17(7):A479. doi: 10.1016/j.jval.2014.08.1381 [90]	Economic	Hypertension	BIA
Khodakarim S FSBELSRVBB. Comparison of sputum conversion time in tuberculosis treatment with fix-dose combination drugs and separate drug regimens. Egyptian journal of chest diseases and tuberculosis 2020;69(3):468. [127]	Clinical	Tuberculosis	Phase 2/3, RCT, open-label, parallel group<br>Phase 2/3, RCT, open-label, parallel group
Koh J-S, Park Y, Tantry US, et al. Pharmacodynamic effects of a new fixed-dose clopidogrel-aspirin combination compared with separate administration of clopidogrel and aspirin in patients treated with coronary stents: The ACCEL-COMBO trial. Platelets 2017;28(2):187-93. doi: https://dx.doi.org/10.1080/09537104.2016.1206197 [60]	Clinical	Coronary stent recovery	Phase not reported, RCT, open-label, single center, 2-way non-inferiority crossover study
Konstas AGP, Katsimpris IE, Kaltsos K, et al. Twenty-four-hour efficacy of the brimonidine/timolol fixed combination versus therapy with the unfixed components. Eye 2008;22(11):1391-97. doi: 10.1038/sj.eye.6702906 [128]	Clinical	Ocular hypertension or primary open-angle glaucoma	Phase not reported, RCT, single-blind, crossover, active-controlled
Kooienga L, Kendrick J, Smits G, et al. The Effect of Combined Calcium and Vitamin D3 Supplementation on Serum Intact Parathyroid Hormone in Moderate CKD. American Journal of Kidney	Clinical	Kidney disease	Phase 3, RCT, double-blind,

Citation	SLR	Indication	Study Design
Diseases 2009;53(3):408-16. doi: http://dx.doi.org/10.1053/j.ajkd.2008.09.020 [129]			comparative, placebo-controlled
Koval SM, Snihurska IO, Starchenko TG, et al. Efficacy of fixed dose of triple combination of perindopril-indapamide-amlodipine in obese patients with moderate-to-severe arterial hypertension: An open-label 6-month study. Biomedical Research and Therapy 2019;6(11):3501-12. doi: http://dx.doi.org/10.15419/bmrat.v6i11.578 [67]	Clinical	Hypertension	Phase not reported, RCT, open-label, parallel-group
Lazcano-Gomez G, Hernandez-Oteyza A, Iriarte-Barbosa MJ, et al. Topical glaucoma therapy cost in Mexico. International Ophthalmology 2014;34(2):241-49. doi: 10.1007/s10792-013-9823-6 [96]	Economic	Glaucoma	Retrospective database analysis
Legorreta A, Yu A, Chernicoff H, et al. Adherence to combined Lamivudine+Zidovudine versus individual components: A community-based retrospective medicaid claims analysis. AIDS Care 2005;17(8):938-48. doi: 10.1080/09540120500100692 [29]	RWE	HIV	Retrospective, database analysis
Levi M, Pasqua A, Cricelli I, et al. Patient Adherence to Olmesartan/Amlodipine Combinations: Fixed Versus Extemporaneous Combinations. Journal of Managed Care & Specialty Pharmacy 2016;22(3):255-62. doi: 10.18553/jmcp.2016.22.3.255 [39]	RWE	Hypertension	Retrospective, database analysis
Machnicki G, Ong SH, Chen W, et al. Comparison of amlodipine/valsartan/hydrochlorothiazide single pill combination and free combination: adherence, persistence, healthcare utilization and costs. Current Medical Research and Opinion 2015;31(12):2287-96. doi: 10.1185/03007995.2015.1098598 [30]	Economic, RWE	Hypertension	Retrospective, database analysis
Maggioni AP, Dondi L, Pedrini A, et al. The use of antiplatelet agents after an acute coronary syndrome in a large community Italian setting of more than 12 million subjects. European Heart Journal Acute Cardiovascular Care 2019;8(6):527-35. doi: 10.1177/2048872618801252 [54]	RWE	Acute coronary syndrome	Retrospective, database analysis
Mariani J, Rosende A, De Abreu M, et al. Multicap to improve adherence after acute coronary syndromes: results of a randomized controlled clinical trial. Therapeutic Advances in Cardiovascular Disease 2020;14 doi: http://dx.doi.org/10.1177/1753944720912071 [61]	Clinical	Myocardial infarction	Phase 3, RCT, parallel-group, open-label, single center
Medin E, Safioti G, Lindqvist F, et al. Updated medication costs from a real-life cost-effectiveness evaluation of budesonide/formoterol maintenance and reliever therapy in asthma maintenance and reliever therapy in asthma [abstract]. ISPOR 18th Annual European Congress 2015;18(7):A500. [97]	Economic	Asthma	Retrospective database analysis
Nazari SS, Fallah S, Raeisi V. Treatment outcome in smear-positive pulmonary tuberculosis patients treated with a fixed-dose drug combination regimen in comparison with a separate regimen: A randomized clinical trial. Egyptian Journal of Chest Diseases and Tuberculosis 2021;70(1):26-30. doi: http://dx.doi.org/10.4103/ejcdt.ejcdt_110_19 [130]	Clinical	Tuberculosis	Phase 2/3, RCT, open-label, parallel group
Nedogoda Sv SVJ. Single-Pill Combination of Perindopril/Indapamide/Amlodipine in Patients with Uncontrolled Hypertension: a Randomized Controlled Trial. Cardiology and therapy 2017;6(1):91. [62]	Clinical	Hypertension	Phase not reported, RCT, open-label, multicenter
Nenasheva N, Nosulya E, Kim I, et al. The efficacy of the fixed combination of mometasone furoate and azelastine hydrochloride as a nasal spray in adult patients with perennial rhinitis. Allergy: European Journal of Allergy and Clinical Immunology 2019;74(Supplement 106):400-01. doi: http://dx.doi.org/10.1111/all.13961 [131]	Clinical	Allergic rhinitis	Phase not reported, RCT, open, parallel-group, multicenter

Citation	SLR	Indication	Study Design
Ofili E, Anand I, Williams RA, et al. Fixed-Dose Versus Off-Label Combination of Isosorbide Dinitrate Plus Hydralazine Hydrochloride: Retrospective Propensity-Matched Analysis in Black Medicare Patients with Heart Failure. Advances in Therapy 2017;34(8):1976-88. doi: 10.1007/s12325-017-0584-x [132]	RWE	Heart failure	Retrospective, database analysis
Olszanecka-Glinianowicz M, Smertka M, Chudek J, et al. Ramipril/amlodipine single pill - Effectiveness, tolerance and patient satisfaction with antihypertensive therapy in relation to nutritional status. Pharmacological Reports 2014;66(6):1043-49. doi: http://dx.doi.org/10.1016/j.pharep.2014.06.020 [75]	HRQoL	Hypertension	Cross-sectional survey
Ong S, Machnicki G, Chen W, et al. Persistence and Adherence with Exforge HCT Single Pill Combination versus Amlodipine/Valsartan/Hydrochlorothiazide Free Combination: A Comparison Controlling for Demographic and Clinical Factors. European Heart Journal 2014;35(suppl_1):1-172. doi: 10.1093/eurheartj/ehu322 [31]	RWE	Hypertension	Retrospective, database analysis
Patel B, Leslie R, Thiebaud P, et al. Adherence with single-pill amlodipine/atorvastatin vs a two-pill regimen. Vascular Health Risk Management 2008;4:673-81. [32]	RWE	Hypertension	Retrospective, database analysis
Perrin K, Williams M, Wijesinghe M, et al. Randomized controlled trial of adherence with single or combination inhaled corticosteroid/long-acting β-agonist inhaler therapy in asthma. Journal of Allergy and Clinical Immunology 2010;126(3):505-10. doi: 10.1016/j.jaci.2010.06.033 [63]	Clinical, HRQoL	Asthma	Phase not reported, RCT, single-blinded, parallel-group, multicenter
Predel H-G, Weisser B, Wassmann S et al. The single pill concept leads to improved persistence of medication, clinical outomes and reduced all-cause mortaility in hypertensive patients - results from the START project. Journal of Hypertension, 39 (2021). [133]	RWE	Cardiovascular disease	Retrospective, database analysis
Predel HG, Weisser B, Wassmann S, et al. Persistence and cardiovascular outcomes with ramipril, atorvastatin, ASA as a single pill compared to the multi pill combination. A subanalysis of the START study, a claims data analysis. European Heart Journal 2020;41(Supplement_2):ehaa946.2964. doi: 10.1093/ehjci/ehaa946.2964 [49]	Economic, RWE	Cardiovascular disease	Retrospective, database analysis
Price D, Keininger D, Costa-Scharplatz M, et al. Cost-effectiveness of the LABA/LAMA dual bronchodilator indacaterol/glycopyrronium in a Swedish healthcare setting. Respiratory Medicine 2014;108(12):1786-93. doi: 10.1016/j.rmed.2014.09.015 [87]	Economic	COPD	Cost minimization analysis
Ren M, Xuan D, Lu Y, et al. Economic evaluation of olmesartan/amlodipine fixed-dose combination for hypertension treatment in China. Journal of Medical Economics 2020;23(4):394-400. doi: 10.1080/13696998.2019.1699799 [88]	Economic	Hypertension	CEA/CUA
Rombopoulos G, Hatzikou M, Athanasiadis A, et al. Treatment Compliance with Fixed-Dose Combination of Vildagliptin/Metformin in Patients with Type 2 Diabetes Mellitus Inadequately Controlled with Metformin Monotherapy: A 24-Week Observational Study. International Journal of Endocrinology 2015;2015:251485. doi: 10.1155/2015/251485 [43]	RWE	Type 2 diabetes mellitus	Prospective, observational, multicenter
Rombopoulos G, Hatzikou M, Kossiva E, et al. Preliminary Results of a Multicenter Observational Study of Treatment Compliance With Free-Combination Versus Fixed Combination Treatment in Type II Diabetes Mellitus Patients in Greece (Less Study). Value in Health 2012;15(7):A503. doi: 10.1016/j.jval.2012.08.1696 [44]	RWE	Type 2 diabetes mellitus	Prospective, Multicenter
Rosenhall L, Borg S, Andersson F, et al. Budesonide/formoterol in a single inhaler (Symbicort) reduces healthcare costs compared with	Economic	Hypertension	Retrospective database analysis

Citation	SLR	Indication	Study Design
separate inhalers in the treatment of asthma over 12 months. International Journal of Clinical Practice 2003;57(8):662-67.			
Rosenhall L, Elvstrand A, Tilling B, et al. One-year safety and efficacy of budesonide/formoterol in a single inhaler (Symbicort Turbuhaler) for the treatment of asthma. Respir Med 2003;97(6):702-8. doi: 10.1053/rmed.2003.1504 [published Online First: 2003/06/20] [134]	Clinical	Asthma	Phase not reported, RCT, open-label, parallel group, multicenter, extension study
Rosenhall L, Heinig J, Lindqvist A, et al. Budesonide/formoterol (Symbicort) is well tolerated and effective in patients with moderate persistent asthma. International Journal of Clinical Practice 2002;56(6):427-33. [81]	Clinical, HRQoL	Asthma	Phase not reported, RCT, open-label, multicenter
Rosso R, Di Biagio A, Maggiolo F, et al. Patient-reported outcomes and low-level residual HIV-RNA in adolescents perinatally infected with HIV-1 after switching to one-pill fixed-dose regimen. AIDS Care 2012;24(1):54-58. doi: 10.1080/09540121.2011.596511 [64]	Clinical, HRQoL	HIV	Phase not reported, single-arm, open-label, crossover, descriptive
Saju S, Varghese F, S D, et al. Pharmacoeconomic Evaluation: Cost Effectiveness Analysis of Oral Antidiabetic Therapy in A Tertiary Care Hospital. International Journal of Pharmaceutical Sciences Review and Research 2021;66:31-37. doi: 10.47583/ijpsrr.2021.v66i01.008 [99]	Economic	Type 2 diabetes mellitus	HCRU
Sandberg A, Kostev K, Ehlken B, Holz B, Oberdiek A. Persistence and compliance in hypertension treatment with olmesartan medoxomil analysis of real-life prescription data. Value in Health, 14(3), A43 (2011). [45]	RWE	Hypertension	Retrospective, database analysis
Simons LA, Chung E, Ortiz M. Long-term persistence with single-pill, fixed-dose combination therapy versus two pills of amlodipine and perindopril for hypertension: Australian experience. Current Medical Research and Opinion 2017;33(10):1783-87. doi: 10.1080/03007995.2017.1367275 [48]	RWE	Hypertension	Retrospective, database analysis
Simons LA, Ortiz M, Calcino G. Persistence with a single pill versus two pills of amlodipine and atorvastatin: the Australian experience, 2006–2010. Medical Journal of Australia 2011;195(3):134-37. doi: https://doi.org/10.5694/j.1326-5377.2011.tb03240.x [50]	RWE	Hypertension	Retrospective, database analysis
Simonyi G, Ferenci T. Persistence of fixed and free combination of ramipril and amlodipine in hypertension. 2015. [51]	RWE	Hypertension	Retrospective, database analysis
Simonyi G, Ferenci T, Medvegy M. Persistence of fixed and free combination of perindopril and Amlodipine in hypertension. European Heart Journal 2016;37(suppl_1):191-598. doi: 10.1093/eurheartj/ehw432 [47]	RWE	Hypertension	Retrospective, database analysis
Simonyi G, Ferenczi T, Medvegy M, et al. Which is the best choice? One year persistence of ramipril, ramipril/amlodipin free and fixed dose combination therapy in hypertension. European Heart Journal 2017;38(suppl_1):ehx502.P1654. doi: 10.1093/eurheartj/ehx502.P1654 [46]	RWE	Hypertension	Retrospective, database analysis
Sproviero E, Albamonte E, Costantino C et al. Efficacy and safety of a fixed combination of intramuscular diclofenac 75 mg + thiocolchicoside 4 mg in the treatment of acute low back pain: a phase III, randomized, double blind, controlled trial. European journal of physical and rehabilitation medicine, 54(5), 654 (2018). [83]	Clinical, HRQoL	Acute lower back pain	Phase 3, RCT, double-blind, parallel-group
Stafylas P, Karaiskou M, Zouka M. Budget impact analysis of the introduction of a single-pill combination of atorvastatin, perindopril and amlodipine in the Greek setting. Value in Health, 21, S99-S100 (2018). [91]	Economic	Hypertension and/or stable coronary artery disease	BIA

Citation	SLR	Indication	Study Design
Stafylas P, Stamuli E, Karaiskou M, Panteris E, Chotzagiannoglou V, Beletsi A. Cost analysis of the introduction of a single-pill combination of rosuvastatin and ezetimibe in the Greek setting. Value in Health, 22, S548 (2019). [92]	Economic	Hyperlipidemia	BIA
Ställberg B, Ekström T, Neij F, et al. A real-life cost-effectiveness evaluation of budesonide/formoterol maintenance and reliever therapy in asthma. Respiratory Medicine 2008;102(10):1360-70. doi: 10.1016/j.rmed.2008.06.017 [76]	Economic, HRQoL	Asthma	HCRU
Stawowczyk E, Holko P, Kawalec P, et al. Cost-Utility Analysis Of Hypertensive Treatment With Indapamide And Amlodipine Single-Pill Combination In The Polish Setting. Value in Health 2014;17(7):A491. doi: 10.1016/j.jval.2014.08.1450 [89]	Economic	Hypertension	CUA
Stempel DA, Stoloff SW, Carranza Rosenzweig JR, et al. Adherence to asthma controller medication regimens. Respiratory Medicine 2005;99(10):1263-67. doi: 10.1016/j.rmed.2005.03.002 [53]	RWE	Asthma	Retrospective, database analysis
Stoloff S, Stempel D, Meyer J, et al. Improved refill persistence with fluticasone propionate and salmeterol in a single inhaler compared with other controller therapies. Journal of Allergy and Clinical Immunology 2004;113(2):245-51. [33]	RWE	Asthma	Retrospective, database analysis
Su WJ, Perng RP. Fixed-dose combination chemotherapy (Rifater/Rifinah) for active pulmonary tuberculosis in Taiwan: a two-year follow-up. The international journal of tuberculosis and lung disease : the official journal of the International Union against Tuberculosis and Lung Disease 2002;6(11):1029-32. [72]	Clinical	Tuberculosis	Phase not reported, RCT
Sung J, Ahn KT, Cho B-R et al. Adherence to triple-component antihypertensive regimens is higher with single-pill than equivalent two-pill regimens: a randomized controlled trial. Clinical and translational science, (2021). [68]	Clinical	Hypertension	Phase not reported, RCT, open-label, parallel group, multicenter
Suryanto AA, van den Broek J, Hatta M, et al. Is there an increased risk of TB relapse in patients treated with fixed-dose combination drugs in Indonesia? The international journal of tuberculosis and lung disease : the official journal of the International Union against Tuberculosis and Lung Disease 2008;12(2):174-9. [135]	Clinical	Tuberculosis	Phase not reported, RCT, long-term follow-up
Thayer S, Arondekar B, Harley C, et al. Adherence to a Fixed-Dose Combination of Rosiglitazone/Glimepiride in Subjects Switching from Monotherapy or Dual Therapy with a Thiazolidinedione and/or a Sulfonylurea. Annals of Pharmacotherapy 2010;44(5):791-99. doi: 10.1345/aph.1M426 [34]	RWE	Type 2 diabetes mellitus	Retrospective, database analysis
Vanderpoel DR, Hussein MA, Watson-Heidari T, et al. Adherence to a fixed-dose combination of rosiglitazone maleate/metformin hydrochloride in subjects with type 2 diabetes mellitus: A retrospective database analysis. Clinical Therapeutics 2004;26(12):2066-75. doi: 10.1016/j.clinthera.2004.12.018 [35]	RWE	HIV	Retrospective, database analysis
Velvanathan T, Islahudin F, Taha NA, et al. Simplification of HAART therapy on ambulatory HIV patients in Malaysia: A randomized controlled trial. Pharmacy Practice 2016;14(4):830. doi: http://dx.doi.org/10.18549/PharmPract.2016.04.830 [82]	Clinical, HRQoL	HIV	Phase not reported, RCT
Vera J, Aragão F, Guimaraes M, et al. Benefits of ART simplification on adherence, clinical and economic outcomes. Journal of the International AIDS Society 2012;15(S4):18064. https://doi.org/10.7448/IAS.15.6.18064 [136]	RWE	HIV	Retrospective, database analysis
Vidaurre Mora EV, Moreno U, Lazcano G, Jiménez Román J. Comparison of the cost of topical therapy for glaucoma between	Economic	Glaucoma	Retrospective, database analysis

Citation	SLR	Indication	Study Design
generic and brand medicines in Mexico. Investigative Ophthalmology & Visual Science, 60(9), 5472-5472 (2019). [100]			
Visco V, Finelli R, Pascale AV, et al. Larger Blood Pressure Reduction by Fixed-Dose Compared to Free Dose Combination Therapy of ACE Inhibitor and Calcium Antagonist in Hypertensive Patients. Transl Med UniSa 2017;16:17-23. [137]	Clinical	Hypertension	Phase not reported, RCT
Zaka-Ur-Rehman Z, Jamshaid M, Chaudhry A. Clinical evaluation and monitoring of adverse effects for fixed multidose combination against single drug therapy in pulmonary tuberculosis patients. Pak J Pharm Sci. 2008;21(2):185-94. [138]	Clinical	Tuberculosis	Phase not reported, RCT, single-center
Zetterström O, Buhl R, Mellem H, et al. Improved asthma control with budesonide/formoterol in a single inhaler, compared with budesonide alone. *Eur Resp J*. 2001;18(2):262. doi:10.1183/09031936.01.00065801 [65]	Clinical, HRQoL	Asthma	Phase not reported, RCT, double-blind, double-dummy, active-controlled, parallel-group design, multicenter
Zhao J-L, Ge J, Li X-X, et al. Comparative efficacy and safety of the fixed versus unfixed combination of latanoprost and timolol in Chinese patients with open-angle glaucoma or ocular hypertension. *BMC Ophthalmol*. 2011;11:23 https://dx.doi.org/10.1186/1471-2415-11-23 [139]	Clinical	Open-angle glaucoma or ocular hypertension	Phase 3, RCT, open-label, parallel-group, multicenter

Abbreviations: BIA, budget impact analysis; CEA, cost-effectiveness analysis; CUA, cost-utility analysis; COPD, chronic obstructive pulmonary disease; HCRU, healthcare resource utilization; HRQoL, health-related quality of life; RCT, randomized clinical trial; RWE, real-world evidence.
